# Supplementary figures and images for: Whole genome sequencing of Yersinia pestis isolates from Central Asian natural plague foci revealed the role of adaptation to different hosts and environmental conditions in shaping specific genotypes
Source: PLoS Negl Trop Dis. 2025 Sep 12;19(9):e0013533. doi: 10.1371/journal.pntd.0013533 (PMC12445494; doi:10.1371/journal.pntd.0013533)

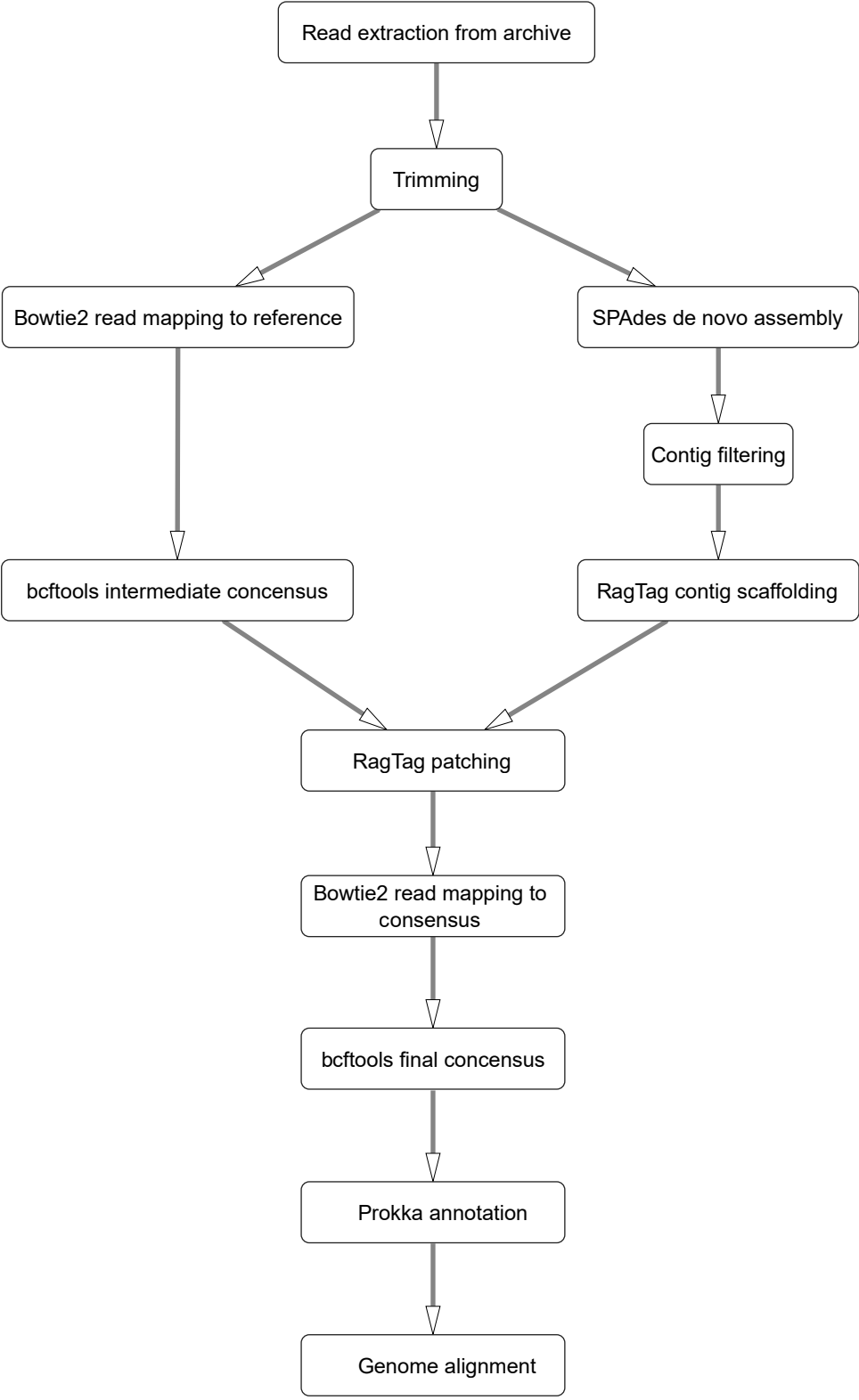

Supplement: S1 Fig — (PDF) [file pntd.0013533.s001.pdf]

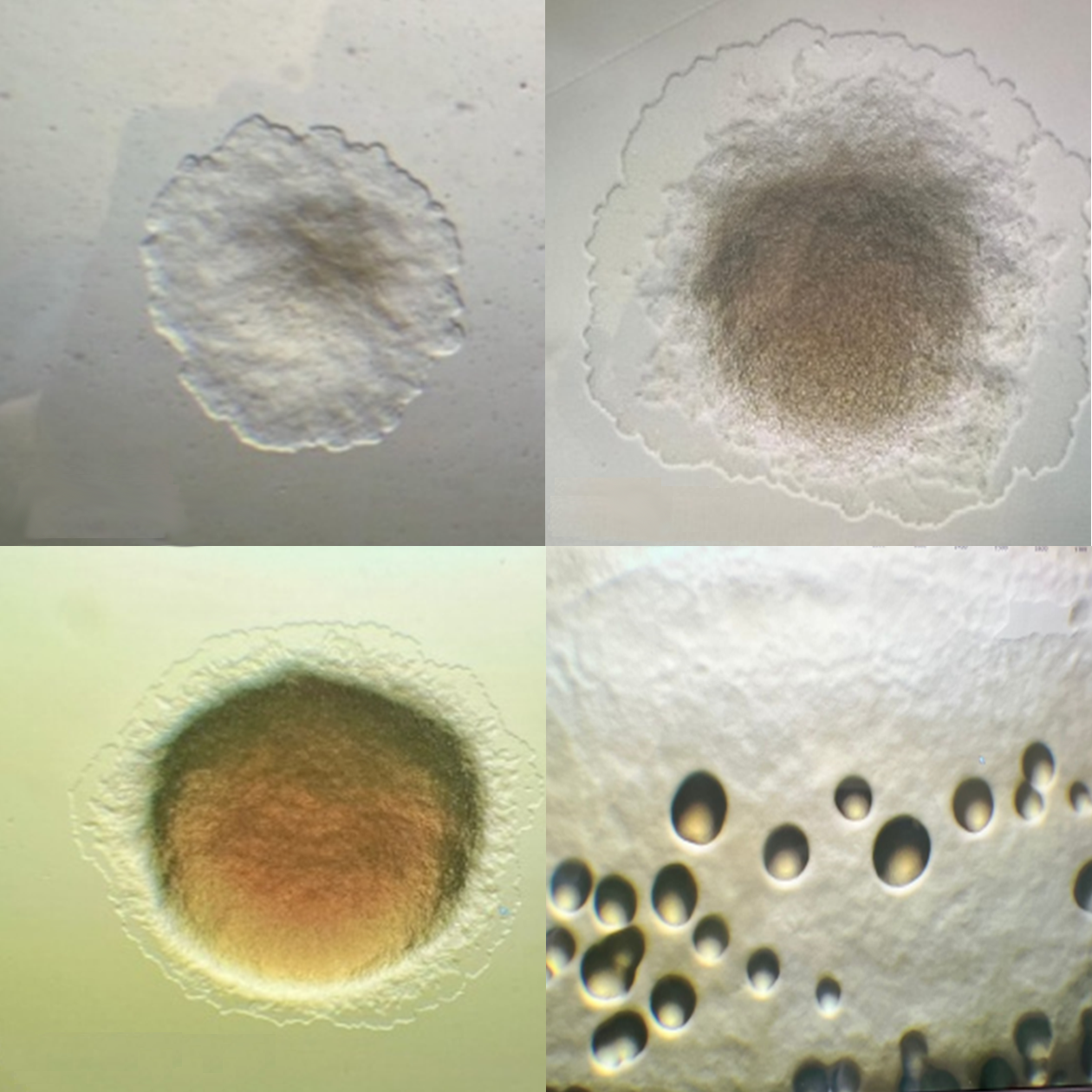

Supplement: S2 Fig — (TIF) [file pntd.0013533.s002.tif]

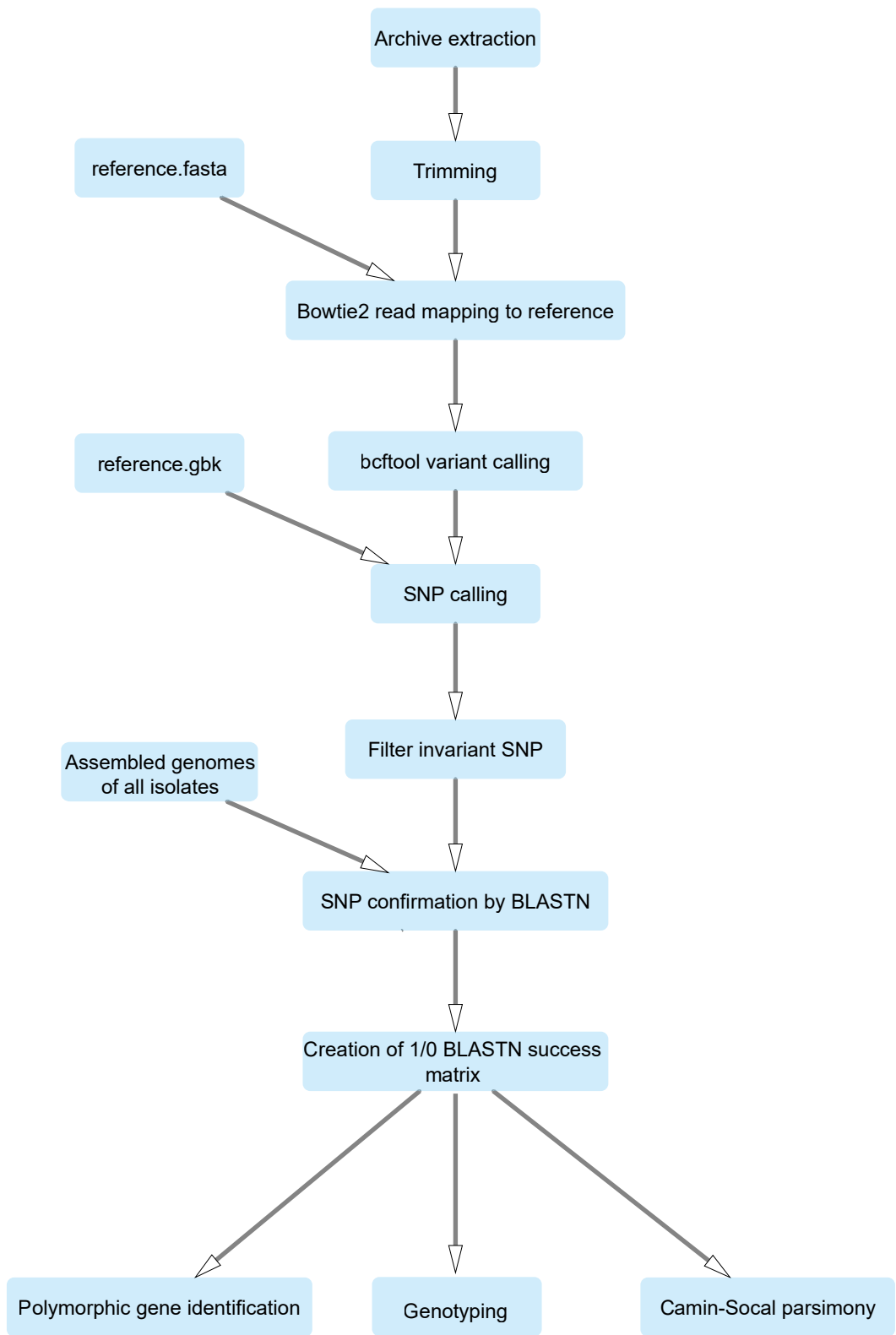

Supplement: S3 Fig — (PDF) [file pntd.0013533.s003.pdf]

Bootstrap Null Distribution  
(Multiclass supported)

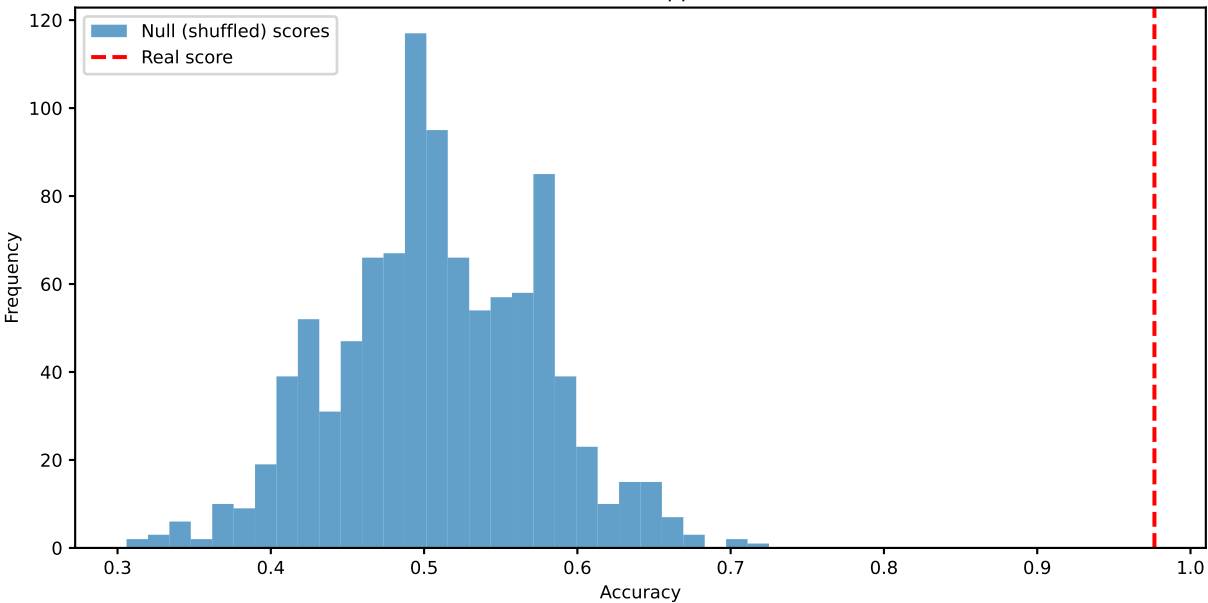

Supplement: S4 Fig — Histogram shows the distribution of classification accuracies obtained from 1,000 Random Forest runs with shuffled group labels (null distribution). The red dashed vertical line indicates the actual classification accuracy 0.9889 achieved using true group labels. The area of the histogram to the right of the left score represents the empirical accuracy classification value, estimating the probability of observing such classification performance by chance. This analysis supports the statistical significance of group separability based on the polymorphic features in the input matrix. (PDF) [file pntd.0013533.s004.pdf]
