# Supplementary material for: Whole genome sequencing of Yersinia pestis isolates from Central Asian natural plague foci revealed the role of adaptation to different hosts and environmental conditions in shaping specific genotypes
Source: PLoS Negl Trop Dis. 2025 Sep 12;19(9):e0013533. doi: 10.1371/journal.pntd.0013533 (PMC12445494; doi:10.1371/journal.pntd.0013533)
Supplement: S2 Table — (DOCX) [file pntd.0013533.s006.docx]

**Supplemental Table S2. Phage susceptibility, biochemical activities and results of diagnostic PCR amplification obtained for selected *Yersinia* isolates.**

| **Strains** | **Phage susceptibility** | | **Fermentation and degradation tests** | | | | **PCR product length** | | | **pCKF detection** |
| --- | --- | --- | --- | --- | --- | --- | --- | --- | --- | --- |
|  | **Pokrovskaya** | **Anti-pseudotuberculosis** | **Glycerin** | **Rhamnose** | **Arabinose** | **Denitrification** | **med24** | ***glpD*** | **ANT/MED** |  |
| 1_YP62_ADT | + | + | + | -- | + | -- | 198 | 508 | 397 | -- |
| 2_YP67_ADT | + | + | + | -- | + | -- | 198 | 508 | 397 | -- |
| 3_YP58_ADT | + | + | + | -- | + | -- | 198 | 508 | 397 | -- |
| 4_YP65_PKK | + | + | + | -- | + | -- | 198 | 508 | 397 | -- |
| 5_YP57_PKK | + | + | + | -- | + | -- | 198 | 508 | 397 | -- |
| 6_YP52_KK | + | + | + | -- | + | -- | 198 | 508 | 397 | -- |
| 7_YP60_IM | + | + | + | -- | + | -- | 198 | 508 | 397 | -- |
| 8_YP55_NP | + | + | + | -- | + | -- | 198 | 508 | 397 | -- |
| 9_YP68_IM | + | + | + | -- | + | -- | 198 | 508 | 397 | -- |
| 10_YP59_IM | + | + | + | -- | + | -- | 198 | 508 | 397 | -- |
| 11_YP51_IM | + | + | + | -- | + | -- | 198 | 508 | 397 | -- |
| 12_YP54_IM | + | + | + | -- | + | -- | 198 | 508 | 397 | -- |
| 13_YP56_IM | + | + | + | -- | + | -- | 198 | 508 | 397 | -- |
| 14_YP75_IM | + | + | + | -- | + | -- | 198 | 508 | 397 | -- |
| 15_YP61_IM | + | + | + | -- | + | -- | 198 | 508 | 397 | -- |
| 16_YP53_IM | + | + | + | -- | + | -- | 198 | 508 | 397 | -- |
| 17_YP69_IM | + | + | + | -- | + | -- | 198 | 508 | 397 | -- |
| 18_YP64_IM | + | + | + | -- | + | -- | 198 | 508 | 397 | -- |
| 19_YP74_IM | + | + | + | -- | + | -- | 198 | 508 | 397 | -- |
| 20_YP71_IM | + | + | + | -- | + | -- | 198 | 508 | 397 | -- |
| 21_YP63_IM | + | + | + | -- | + | -- | 198 | 508 | 397 | -- |
| 22_YP66_IM | + | + | + | -- | + | -- | 198 | 508 | 397 | -- |
| 23_YP72_IM | + | + | + | -- | + | -- | 198 | 508 | 397 | -- |
| 24_YP70_IM | + | + | + | -- | + | -- | 198 | 508 | 397 | -- |
| 25_YP73_IM | + | + | + | -- | + | -- | 198 | 508 | 397 | -- |
| 26_YP30_SZ | + | + | + | -- | + | + | 222 | 508 | 467 | -- |
| 27_YP31_SZ | + | + | + | -- | + | + | 222 | 508 | 467 | -- |
| 28_YP29_SZ | + | + | + | -- | + | + | 222 | 508 | 467 | -- |
| 29_YP8_KK | + | + | + | -- | + | -- | 198 | 508 | 397 | -- |
| 30_YP17_PB | + | + | + | -- | + | -- | 198 | 508 | 397 | -- |
| 31_YP22_SZ | + | + | + | -- | + | + | 222 | 508 | 467 | -- |
| 32_YP32_MOK | + | + | + | -- | + | -- | 198 | 508 | 397 | -- |
| 33_YP16_UE | + | + | + | -- | + | -- | 198 | 508 | 397 | -- |
| 34_YP28_SZ | + | + | + | -- | + | + | 222 | 508 | 467 | -- |
| 35_YP14_TLH | + | + | + | + | -- | -- | 222 | 508 | 467 | -- |
| 36_YP27_TLH | + | + | + | + | -- | -- | 222 | 508 | 467 | -- |
| 37_YP35_TLH | + | + | + | + | -- | -- | 222 | 508 | 467 | -- |
| 38 YP25_SZ | + | + | + | -- | + | + | 222 | 508 | 467 | -- |
| 39_YP13_TLH | + | + | + | + | -- | -- | 222 | 508 | 467 | -- |
| 40_YP4_PB | + | + | + | -- | + | -- | 198 | 508 | 397 | -- |
| 41_YP26_PAK | + | + | + | -- | + | -- | 198 | 508 | 397 | -- |
| 42_YP10_UE | + | + | + | -- | + | -- | 222 | 508 | 397 | + |
| 43_YP6_UE | + | + | + | -- | + | -- | 198 | 508 | 397 | -- |
| 44_YP7_NP | + | + | + | -- | + | -- | 198 | 508 | 397 | -- |
| 45_YP8_PAK | + | + | + | -- | + | -- | 198 | 508 | 397 | -- |
| 46_YP29_PAK | + | + | + | -- | + | -- | 198 | 508 | 397 | -- |
| 47_YP36_PAK | + | + | + | -- | + | -- | 198 | 508 | 397 | -- |
| 48_YP14_PAK | + | + | + | -- | + | -- | 198 | 508 | 397 | -- |
| 49_YP5_PAK | + | + | + | -- | + | -- | 198 | 508 | 397 | -- |
| 50_YP23_PB | + | + | + | -- | + | -- | 198 | 508 | 397 | -- |
| 51_YP1_PAK | + | + | + | -- | + | -- | 198 | 508 | 397 | -- |
| 52_YP18_PAK | + | + | + | -- | + | -- | 198 | 508 | 397 | -- |
| 53_YP3_IM | -- | + | + | + | + | nd | 222 | 508 | 467 | -- |
| 54_YP2_IM | + | + | + | -- | + | -- | 198 | 508 | 397 | -- |
| 55_YP20_MOK | + | + | + | -- | + | -- | 198 | 508 | 397 | -- |
| 56_YP12_TK | + | + | + | -- | + | -- | 198 | 508 | 397 | -- |
| 57_YP22_IM | + | + | + | -- | + | -- | 198 | 508 | 397 | -- |
| 58_YP9_TK | + | + | + | -- | + | -- | 198 | 508 | 397 | -- |
| 59_YP21_IM | + | + | + | -- | + | -- | 198 | 508 | 397 | -- |
| 60_YP24_IM | + | + | + | -- | + | -- | 198 | 508 | 397 | -- |
| 61_YP3_PB | + | + | + | -- | + | -- | 198 | 508 | 397 | -- |
| 62_YP33_PB | + | + | + | -- | + | -- | 198 | 508 | 397 | -- |
| 63_YP2_PB | + | + | + | -- | + | -- | 198 | 508 | 397 | -- |
| 64_YP37_KK | + | + | + | -- | + | -- | 198 | 508 | 397 | -- |
| 65_YP4_KK | + | + | + | -- | + | -- | 198 | 508 | 397 | -- |
| 66_YP38_PB | + | + | + | -- | + | -- | 198 | 508 | 397 | -- |
| 67_YP34_PB | + | + | + | -- | + | -- | 198 | 508 | 397 | -- |
| 68_YP15_PB | + | + | + | -- | + | -- | 198 | 508 | 397 | -- |
| 69_YP30_ADT | + | + | + | -- | + | -- | 198 | 508 | 397 | -- |
| 70_YP27_IM | + | + | + | -- | + | -- | 198 | 508 | 397 | -- |
| 71_YP7_IM | + | + | + | -- | + | -- | 198 | 508 | 397 | -- |
| 72_YP5_PKK | + | + | + | -- | + | -- | 198 | 508 | 397 | -- |
| 73_YP21_KK | + | + | + | -- | + | -- | 198 | 508 | 397 | -- |
| 74_YP19_KK | + | + | + | -- | + | -- | 198 | 508 | 397 | -- |
| 75_YP26_PB | + | + | + | -- | + | -- | 198 | 508 | 397 | -- |
| 76_YP20_MOK | + | + | + | -- | + | -- | 198 | 508 | 397 | -- |
| 77_YP25_IM | + | + | + | -- | + | -- | 198 | 508 | 397 | -- |
| 78_YP23_IM | + | + | + | -- | + | -- | 198 | 508 | 397 | -- |
| 79_YP9_MOK | + | + | + | -- | + | -- | 198 | 508 | 397 | -- |
| 80_YP4_IM | + | + | + | -- | + | -- | 198 | 508 | 397 | -- |
| 81_YP39_IM | + | + | + | -- | + | -- | 198 | 508 | 397 | -- |
| 82_YP1_IM | + | + | + | -- | + | -- | 198 | 508 | 397 | -- |
| 83_YP13_ADT | + | + | + | -- | + | -- | 198 | 508 | 397 | -- |
| 84_YP17_ADT | + | + | + | -- | + | -- | 198 | 508 | 397 | -- |
| 85_YP3_ADT | + | + | + | -- | + | -- | 198 | 508 | 397 | -- |
| 86_YP12_ADT | + | + | + | -- | + | -- | 198 | 508 | 397 | -- |
| 87_YP18_PKK | + | + | + | -- | + | -- | 198 | 508 | 397 | -- |
| 88_YP10_ADT | + | + | + | -- | + | -- | 198 | 508 | 397 | -- |
| 89_YP5_ADT | + | + | + | -- | + | -- | 198 | 508 | 397 | -- |
| 90_YP32_ADT | + | + | + | -- | + | -- | 198 | 508 | 397 | -- |
| 91_YP6_ADT | + | + | + | -- | + | -- | 198 | 508 | 397 | -- |
| 92_YP31_KK | + | + | + | -- | + | -- | 198 | 508 | 397 | -- |
| 93_YP24_KK | + | + | + | -- | + | -- | 198 | 508 | 397 | -- |
| 94_YP6_KK | + | + | + | -- | + | -- | 198 | 508 | 397 | -- |
| 95_YP11_KK | + | + | + | -- | + | -- | 198 | 508 | 397 | -- |
| 96_YP2_KK | + | + | + | -- | + | -- | 198 | 508 | 397 | -- |
| 97_YP1_KK | + | + | + | -- | + | -- | 198 | 508 | 397 | -- |
| 98_YP8_KK | + | + | + | -- | + | -- | 198 | 508 | 397 | -- |
