# Supplementary material for: Whole genome sequencing of Yersinia pestis isolates from Central Asian natural plague foci revealed the role of adaptation to different hosts and environmental conditions in shaping specific genotypes
Source: PLoS Negl Trop Dis. 2025 Sep 12;19(9):e0013533. doi: 10.1371/journal.pntd.0013533 (PMC12445494; doi:10.1371/journal.pntd.0013533)
Supplement: S4 Table — (DOCX) [file pntd.0013533.s008.docx]

Supplemental Table S4. Results of computer-based genotyping of the isolates by allelic states of **classical marker genes and VNTR microsatellite spots.**

| **Strain** | ***ilvN* deletion** | ***araC* deletion** | ***napA* deletion** | **Allelic states at *rhaS*:671** | ***yeaW* polymor-phism** | **Spacer length between Med24 primers** | **Spacer length between ms06 primers** | **Preliminary biovar identification** |
| --- | --- | --- | --- | --- | --- | --- | --- | --- |
| IP 32953 | **–** | **–** | **–** | A | **–** | 230 | 1088 | *Y. pseudotu-berculosis,* reference |
| SCPM-O-B-6899 | **+** | **–** | **–** | G | **–** | 230 | 547 | Central-Caucasus focus, reference |
| 1_YP62_ADT | **+** | **–** | + | A | + | 207 | N | MED |
| 2_YP67_ADT | **+** | **–** | + | A | + | 232 | N | MED |
| 3_YP58_ADT | **+** | **–** | + | A | + | 207 | N | MED |
| 4_YP65_PKK | **+** | **–** | + | A | + | 207 | N | MED |
| 5_YP57_PKK | **+** | **–** | + | A | + | 207 | N | MED |
| 6_YP52_KK | **+** | **–** | + | A | + | 207 | N | MED |
| 7_YP60_IM | **+** | **–** | + | A | + | 232 | N | MED |
| 8_YP55_NP | **+** | **–** | + | A | + | 232 | N | MED |
| 9_YP68_IM | **+** | **–** | + | A | + | 207 | N | MED |
| 10_YP59_IM | **+** | **–** | + | A | + | 207 | N | MED |
| 11_YP51_IM | **+** | **–** | + | A | + | 207 | N | MED |
| 12_YP54_IM | **+** | **–** | + | A | + | 231 | N | MED |
| 13_YP56_IM | **+** | **–** | + | A | + | 207 | N | MED |
| 14_YP75_IM | **+** | **–** | + | A | + | 232 | N | MED |
| 15_YP61_IM | **+** | **–** | + | A | + | 232 | N | MED |
| 16_YP53_IM | **+** | **–** | + | A | + | 207 | N | MED |
| 17_YP69_IM | **+** | **–** | + | A | + | 207 | N | MED |
| 18_YP64_IM | **+** | **–** | + | A | + | 207 | N | MED |
| 19_YP74_IM | **+** | **–** | + | A | **–** | 207 | 1094 | Unidentified |
| 20_YP71_IM | **+** | **–** | + | A | + | 207 | N | MED |
| 21_YP63_IM | **+** | **–** | + | A | + | 207 | N | MED |
| 22_YP66_IM | **+** | **–** | + | A | + | 207 | N | MED |
| 23_YP72_IM | **+** | **–** | + | A | + | 207 | N | MED |
| 24_YP70_IM | **+** | **–** | + | A | + | 232 | N | MED |
| 25_YP73_IM | **+** | **–** | + | A | + | 207 | N | MED |
| 26_YP30_SZ | **+** | **–** | **–** | A | **–** | 231 | N | ANT |
| 27_YP31_SZ | **+** | **–** | **–** | A | **–** | 231 | N | ANT |
| 28_YP29_SZ | **+** | **–** | **–** | A | **–** | 231 | N | ANT |
| 29_YP8_KK | **+** | **–** | + | A | + | 207 | N | MED |
| 30_YP17_PB | **+** | **–** | + | A | + | 207 | N | MED |
| 31_YP22_SZ | **+** | **–** | **–** | A | **–** | 231 | N | ANT |
| 32_YP32_MOK | **+** | **–** | + | A | + | 207 | N | MED |
| 33_YP16_UE | **+** | **–** | + | A | + | 207 | N | MED |
| 34_YP28_SZ | **+** | **–** | **–** | A | **–** | 231 | N | ANT |
| 35_YP14_TLH | **–** | **+** | **–** | G | **–** | 231 | N | Talas |
| 36_YP27_TLH | **–** | **+** | **–** | G | **–** | 231 | 1090 | Unidentified |
| 37_YP35_TLH | **–** | **+** | **–** | G | **–** | 231 | N | Talas |
| 38 YP25_SZ | **+** | **–** | **–** | A | **–** | 231 | N | ANT |
| 39_YP13_TLH | **–** | **+** | **–** | G | **–** | 231 | N | Talas |
| 40_YP4_PB | **+** | **–** | + | A | + | 207 | N | MED |
| 41_YP26_PAK | **+** | **–** | + | A | + | 207 | N | MED |
| 42_YP10_UE | **+** | **–** | + | A | + | 207 | N | MED |
| 43_YP6_UE | **+** | **–** | + | A | + | 207 | N | MED |
| 44_YP7_NP | **+** | **–** | + | A | + | 207 | N | MED |
| 45_YP8_PAK | **+** | **–** | + | A | + | 207 | N | MED |
| 46_YP29_PAK | **+** | **–** | + | A | + | 207 | N | MED |
| 47_YP36_PAK | **+** | **–** | + | A | + | 207 | N | MED |
| 48_YP14_PAK | **+** | **–** | + | A | + | 207 | N | MED |
| 49_YP5_PAK | **+** | **–** | + | A | + | 207 | 400 | Unidentified |
| 50_YP23_PB | **+** | **–** | + | A | + | 207 | N | MED |
| 51_YP1_PAK | **+** | **–** | + | A | + | 207 | N | MED |
| 52_YP18_PAK | **+** | **–** | + | A | + | 207 | N | MED |
| 53_YP3_IM | **–** | **–** | **–** | G | **–** | 233 | ~1000 | Unidentified |
| 54_YP2_IM | **+** | **–** | + | A | + | 207 | N | MED |
| 55_YP20_MOK | **+** | **–** | + | A | + | 207 | N | MED |
| 56_YP12_TK | **+** | **–** | + | A | + | 207 | N | MED |
| 57_YP22_IM | **–** | **–** | + | A | **–** | 231 | 1090 | Unidentified |
| 58_YP9_TK | **+** | **–** | + | A | + | 207 | N | MED |
| 59_YP21_IM | **+** | **–** | + | A | + | 207 | N | MED |
| 60_YP24_IM | **+** | **–** | + | A | + | 207 | N | MED |
| 61_YP3_PB | **+** | **–** | + | A | + | 207 | N | MED |
| 62_YP33_PB | **+** | **–** | + | A | + | 207 | N | MED |
| 63_YP2_PB | **+** | **–** | + | A | + | 207 | N | MED |
| 64_YP37_KK | **+** | **–** | + | A | + | 207 | N | MED |
| 65_YP4_KK | **+** | **–** | + | A | + | 207 | N | MED |
| 66_YP38_PB | **+** | **–** | + | A | + | 232 | N | MED |
| 67_YP34_PB | **+** | **–** | + | A | + | 207 | N | MED |
| 68_YP15_PB | **+** | **–** | + | A | + | 207 | N | MED |
| 69_YP30_ADT | **+** | **–** | + | A | + | 207 | N | MED |
| 70_YP27_IM | **+** | **–** | + | A | + | 207 | N | MED |
| 71_YP7_IM | **+** | **–** | + | A | + | 207 | N | MED |
| 72_YP5_PKK | **+** | **–** | + | A | + | 207 | N | MED |
| 73_YP21_KK | **+** | **–** | + | A | + | 207 | N | MED |
| 74_YP19_KK | **+** | **–** | + | A | + | 207 | N | MED |
| 75_YP26_PB | **+** | **–** | + | A | + | 207 | N | MED |
| 76_YP20_MOK | **+** | **–** | + | A | + | 207 | N | MED |
| 77_YP25_IM | **+** | **–** | + | A | + | 207 | N | MED |
| 78_YP23_IM | **+** | **–** | + | A | + | 207 | N | MED |
| 79_YP9_MOK | **+** | **–** | + | A | + | 207 | N | MED |
| 80_YP4_IM | **+** | **–** | + | A | + | 207 | N | MED |
| 81_YP39_IM | **+** | **–** | + | A | + | 207 | N | MED |
| 82_YP1_IM | **+** | **–** | + | A | + | 207 | N | MED |
| 83_YP13_ADT | **+** | **–** | + | A | + | 207 | N | MED |
| 84_YP17_ADT | **+** | **–** | + | A | + | 207 | N | MED |
| 85_YP3_ADT | **+** | **–** | + | A | + | 207 | N | MED |
| 86_YP12_ADT | **+** | **–** | + | A | + | 207 | N | MED |
| 87_YP18_PKK | **+** | **–** | + | A | + | 207 | N | MED |
| 88_YP10_ADT | **+** | **–** | + | A | + | 207 | N | MED |
| 89_YP5_ADT | **+** | **–** | + | A | + | 207 | N | MED |
| 90_YP32_ADT | **+** | **–** | + | A | + | 207 | N | MED |
| 91_YP6_ADT | **+** | **–** | + | A | + | 207 | N | MED |
| 92_YP31_KK | **+** | **–** | + | A | + | 207 | N | MED |
| 93_YP24_KK | **+** | **–** | + | A | + | 207 | N | MED |
| 94_YP6_KK | **+** | **–** | + | A | + | 207 | N | MED |
| 95_YP11_KK | **+** | **–** | + | A | + | 207 | N | MED |
| 96_YP2_KK | **+** | **–** | + | A | + | 207 | N | MED |
| 97_YP1_KK | **+** | **–** | + | A | + | 207 | N | MED |
| 98_YP8_KK | **+** | **–** | + | A | + | 207 | N | MED |
