# Supplementary material for: Whole genome sequencing of Yersinia pestis isolates from Central Asian natural plague foci revealed the role of adaptation to different hosts and environmental conditions in shaping specific genotypes
Source: PLoS Negl Trop Dis. 2025 Sep 12;19(9):e0013533. doi: 10.1371/journal.pntd.0013533 (PMC12445494; doi:10.1371/journal.pntd.0013533)
Supplement: S6 Table — (DOCX) [file pntd.0013533.s010.docx]

Supplemental Table S6. Numbers of transposons and CRISPR-Cas elements identified in different *Yersinia* strains.

| **Isolates** | **Clade** | **IS630 Family** | | | **IS3 Family** | | | | | | | | **IS5 Family** | | | **IS21 Family** | **IS256 Family** | | | **IS481 Family** | | **IS1595 Family** | **Tn3 Family** | **Tn totals** | **CRISPR-Cas** |
| --- | --- | --- | --- | --- | --- | --- | --- | --- | --- | --- | --- | --- | --- | --- | --- | --- | --- | --- | --- | --- | --- | --- | --- | --- | --- |
|  |  | **IS100** | **IS100kyp** | **IS285** | **IS1222** | **IS1400** | **IS1661** | **ISPa31** | **ISPa74** | **ISYps8** | **ISYpe1** | **ISVisp4** | **IS1N** | **IS1541B** | **IS200G** | **IS903** | **ISYps7** | **ISRor2** | **ISSsu9** | **ISEc39** | **ISEc62** | **ISVsa5** | **TnXax1** |  |  |
| SCPM-O-B-6899 | NC | 1 | 29 | 1 | 1 | 1 | 8 | 1 | 1 | 2 | 0 | 0 | 1 | 33 | 28 | 1 | 3 | 1 | 1 | 22 | 0 | 0 | 0 | 135 | 3 |
| IP 32953 | YPT | 0 | 5 | 1 | 0 | 2 | 3 | 1 | 3 | 1 | 1 | 0 | 1 | 4 | 1 | 0 | 3 | 1 | 1 | 7 | 1 | 2 | 0 | 38 | 2 |
| 53_YP3_IM | YPT | 0 | 5 | 1 | 0 | 2 | 3 | 1 | 3 | 2 | 0 | 0 | 1 | 4 | 1 | 0 | 3 | 1 | 1 | 7 | 1 | 2 | 0 | 38 | 4 |
| 31_YP22_SZ | ANT | 1 | 28 | 2 | 1 | 1 | 8 | 1 | 1 | 1 | 1 | 0 | 1 | 34 | 29 | 1 | 3 | 1 | 1 | 20 | 0 | 0 | 0 | 135 | 2 |
| 34_YP28_SZ | ANT | 1 | 28 | 2 | 1 | 1 | 8 | 1 | 1 | 1 | 1 | 0 | 1 | 34 | 29 | 1 | 3 | 1 | 1 | 20 | 0 | 0 | 0 | 135 | 2 |
| 38 YP25_SZ | ANT | 0 | 28 | 2 | 1 | 1 | 8 | 1 | 1 | 1 | 1 | 0 | 1 | 34 | 29 | 1 | 3 | 1 | 1 | 20 | 0 | 0 | 0 | 134 | 2 |
| 26_YP30_SZ | ANT | 1 | 28 | 1 | 1 | 1 | 8 | 1 | 1 | 2 | 0 | 0 | 1 | 34 | 29 | 1 | 3 | 1 | 1 | 21 | 0 | 0 | 0 | 135 | 2 |
| 27_YP31_SZ | ANT | 1 | 28 | 1 | 1 | 1 | 8 | 1 | 1 | 1 | 1 | 0 | 1 | 32 | 31 | 1 | 3 | 1 | 1 | 21 | 0 | 0 | 0 | 135 | 2 |
| 28_YP29_SZ | ANT | 2 | 28 | 1 | 1 | 1 | 8 | 1 | 1 | 2 | 0 | 0 | 1 | 34 | 29 | 1 | 3 | 1 | 1 | 21 | 0 | 0 | 0 | 136 | 2 |
| 1_YP62_ADT | MED-D | 2 | 28 | 2 | 1 | 1 | 8 | 1 | 1 | 1 | 1 | 0 | 1 | 34 | 29 | 1 | 3 | 1 | 1 | 20 | 0 | 0 | 0 | 136 | 2 |
| 2_YP67_ADT | MED-D | 2 | 28 | 2 | 1 | 1 | 8 | 1 | 1 | 1 | 1 | 0 | 1 | 34 | 29 | 1 | 3 | 1 | 1 | 20 | 0 | 0 | 0 | 136 | 2 |
| 29_YP8_KK | MED-D | 0 | 28 | 2 | 1 | 1 | 8 | 1 | 1 | 1 | 1 | 0 | 1 | 33 | 30 | 1 | 3 | 1 | 1 | 20 | 0 | 0 | 0 | 134 | 3 |
| 3_YP58_ADT | MED-D | 2 | 28 | 2 | 1 | 1 | 8 | 1 | 1 | 1 | 1 | 0 | 1 | 34 | 29 | 1 | 3 | 1 | 1 | 20 | 0 | 0 | 0 | 136 | 2 |
| 30_YP17_PB | MED-D | 1 | 28 | 1 | 1 | 1 | 8 | 1 | 1 | 1 | 1 | 0 | 1 | 36 | 27 | 1 | 3 | 1 | 1 | 21 | 0 | 0 | 0 | 135 | 2 |
| 32_YP32_MOK | MED-D | 1 | 28 | 2 | 1 | 1 | 8 | 1 | 1 | 1 | 1 | 0 | 1 | 32 | 31 | 1 | 3 | 1 | 1 | 20 | 0 | 0 | 0 | 135 | 2 |
| 33_YP16_UE | MED-D | 1 | 28 | 2 | 1 | 1 | 8 | 1 | 1 | 1 | 1 | 0 | 1 | 34 | 29 | 1 | 3 | 1 | 1 | 20 | 0 | 0 | 0 | 135 | 2 |
| 5_YP57_PKK | MED-D | 2 | 28 | 2 | 1 | 1 | 8 | 1 | 1 | 1 | 1 | 0 | 1 | 34 | 29 | 1 | 3 | 1 | 1 | 20 | 0 | 0 | 0 | 136 | 1 |
| 54_YP2_IM | MED-D | 1 | 28 | 2 | 1 | 1 | 8 | 1 | 1 | 1 | 1 | 0 | 1 | 32 | 31 | 1 | 3 | 1 | 1 | 20 | 0 | 0 | 0 | 135 | 3 |
| 55_YP20_MOK | MED-D | 1 | 28 | 2 | 1 | 1 | 8 | 1 | 1 | 1 | 1 | 0 | 1 | 34 | 29 | 1 | 3 | 1 | 1 | 20 | 0 | 0 | 0 | 135 | 2 |
| 6_YP52_KK | MED-D | 2 | 28 | 2 | 1 | 1 | 8 | 1 | 1 | 1 | 1 | 0 | 1 | 34 | 29 | 1 | 3 | 1 | 1 | 20 | 0 | 0 | 0 | 136 | 2 |
| 64_YP37_KK | MED-D | 2 | 28 | 2 | 1 | 1 | 8 | 1 | 1 | 1 | 1 | 0 | 1 | 35 | 28 | 1 | 3 | 1 | 1 | 20 | 0 | 0 | 0 | 136 | 3 |
| 65_YP4_KK | MED-D | 1 | 28 | 2 | 1 | 1 | 8 | 1 | 1 | 1 | 1 | 0 | 1 | 33 | 30 | 1 | 3 | 1 | 1 | 20 | 0 | 0 | 0 | 135 | 2 |
| 68_YP15_PB | MED-D | 2 | 28 | 2 | 1 | 1 | 8 | 1 | 1 | 1 | 1 | 0 | 1 | 33 | 30 | 1 | 3 | 1 | 1 | 20 | 0 | 0 | 0 | 136 | 2 |
| 69_YP30_ADT | MED-D | 1 | 28 | 2 | 1 | 1 | 8 | 1 | 1 | 1 | 1 | 0 | 1 | 34 | 29 | 1 | 3 | 1 | 1 | 20 | 0 | 0 | 0 | 135 | 2 |
| 72_YP5_PKK | MED-D | 1 | 28 | 2 | 1 | 1 | 8 | 1 | 1 | 1 | 1 | 0 | 1 | 35 | 28 | 1 | 3 | 1 | 1 | 20 | 0 | 0 | 0 | 135 | 2 |
| 74_YP19_KK | MED-D | 1 | 28 | 2 | 1 | 1 | 8 | 1 | 1 | 1 | 1 | 0 | 1 | 36 | 27 | 1 | 3 | 1 | 1 | 20 | 0 | 0 | 0 | 135 | 2 |
| 76_YP20_MOK | MED-D | 1 | 28 | 2 | 1 | 1 | 8 | 1 | 1 | 1 | 1 | 0 | 1 | 33 | 30 | 1 | 3 | 1 | 1 | 20 | 0 | 0 | 0 | 135 | 2 |
| 78_YP23_IM | MED-D | 1 | 28 | 2 | 1 | 1 | 8 | 1 | 1 | 1 | 1 | 0 | 1 | 35 | 28 | 1 | 3 | 1 | 1 | 20 | 0 | 0 | 0 | 135 | 2 |
| 79_YP9_MOK | MED-D | 1 | 28 | 2 | 1 | 1 | 8 | 1 | 1 | 1 | 1 | 0 | 1 | 32 | 31 | 1 | 3 | 1 | 1 | 20 | 0 | 0 | 0 | 135 | 3 |
| 8_YP55_NP | MED-D | 2 | 28 | 2 | 1 | 1 | 8 | 1 | 1 | 1 | 1 | 0 | 1 | 34 | 29 | 1 | 3 | 1 | 1 | 20 | 0 | 0 | 1 | 137 | 2 |
| 80_YP4_IM | MED-D | 2 | 28 | 2 | 1 | 1 | 8 | 1 | 1 | 1 | 1 | 0 | 1 | 34 | 29 | 1 | 3 | 1 | 1 | 20 | 0 | 0 | 0 | 136 | 2 |
| 84_YP17_ADT | MED-D | 1 | 28 | 2 | 1 | 1 | 8 | 1 | 1 | 1 | 1 | 0 | 1 | 35 | 28 | 1 | 3 | 1 | 1 | 20 | 0 | 0 | 0 | 135 | 2 |
| 85_YP3_ADT | MED-D | 1 | 28 | 2 | 1 | 1 | 8 | 1 | 1 | 1 | 1 | 0 | 1 | 34 | 29 | 1 | 3 | 1 | 1 | 20 | 0 | 0 | 0 | 135 | 1 |
| 86_YP12_ADT | MED-D | 1 | 28 | 1 | 1 | 1 | 8 | 1 | 1 | 1 | 1 | 0 | 1 | 34 | 29 | 1 | 3 | 1 | 1 | 21 | 0 | 0 | 0 | 135 | 2 |
| 87_YP18_PKK | MED-D | 2 | 28 | 2 | 1 | 1 | 8 | 1 | 1 | 1 | 1 | 0 | 1 | 35 | 28 | 1 | 3 | 1 | 1 | 20 | 0 | 0 | 0 | 136 | 2 |
| 88_YP10_ADT | MED-D | 2 | 28 | 2 | 1 | 1 | 8 | 1 | 1 | 1 | 1 | 0 | 1 | 32 | 31 | 1 | 3 | 1 | 1 | 20 | 0 | 0 | 0 | 136 | 2 |
| 89_YP5_ADT | MED-D | 1 | 28 | 2 | 1 | 1 | 8 | 1 | 1 | 1 | 1 | 0 | 1 | 35 | 28 | 1 | 3 | 1 | 1 | 20 | 0 | 0 | 0 | 135 | 2 |
| 90_YP32_ADT | MED-D | 2 | 28 | 2 | 1 | 1 | 8 | 1 | 1 | 1 | 1 | 0 | 1 | 34 | 29 | 1 | 3 | 1 | 1 | 20 | 0 | 0 | 0 | 136 | 2 |
| 91_YP6_ADT | MED-D | 1 | 28 | 2 | 1 | 1 | 8 | 1 | 1 | 1 | 1 | 0 | 1 | 34 | 29 | 1 | 3 | 1 | 1 | 20 | 0 | 0 | 0 | 135 | 2 |
| 92_YP31_KK | MED-D | 2 | 28 | 2 | 1 | 1 | 8 | 1 | 1 | 1 | 1 | 0 | 1 | 34 | 29 | 1 | 3 | 1 | 1 | 20 | 0 | 0 | 0 | 136 | 1 |
| 94_YP6_KK | MED-D | 1 | 28 | 1 | 1 | 1 | 8 | 1 | 1 | 1 | 1 | 0 | 1 | 34 | 29 | 1 | 3 | 1 | 1 | 21 | 0 | 0 | 0 | 135 | 1 |
| 95_YP11_KK | MED-D | 0 | 28 | 2 | 1 | 1 | 8 | 1 | 1 | 1 | 1 | 0 | 1 | 33 | 30 | 1 | 3 | 1 | 1 | 20 | 0 | 0 | 0 | 134 | 2 |
| 96_YP2_KK | MED-D | 1 | 28 | 2 | 1 | 1 | 8 | 1 | 1 | 1 | 1 | 0 | 1 | 34 | 29 | 1 | 3 | 1 | 1 | 20 | 0 | 0 | 0 | 135 | 2 |
| 97_YP1_KK | MED-D | 0 | 28 | 2 | 1 | 1 | 8 | 1 | 1 | 1 | 1 | 0 | 1 | 32 | 31 | 1 | 3 | 1 | 1 | 20 | 0 | 0 | 0 | 134 | 2 |
| 98_YP8_KK | MED-D | 0 | 28 | 2 | 1 | 1 | 8 | 1 | 1 | 1 | 1 | 0 | 1 | 32 | 31 | 1 | 3 | 1 | 1 | 20 | 0 | 0 | 0 | 134 | 2 |
| 42_YP10_UE | MED-D | 2 | 28 | 2 | 1 | 1 | 8 | 1 | 1 | 1 | 1 | 0 | 1 | 33 | 30 | 1 | 3 | 1 | 1 | 20 | 0 | 0 | 0 | 136 | 1 |
| 10_YP59_IM | MED-U | 1 | 28 | 2 | 1 | 1 | 8 | 1 | 1 | 1 | 1 | 0 | 1 | 34 | 29 | 1 | 3 | 1 | 1 | 20 | 0 | 0 | 0 | 135 | 2 |
| 11_YP51_IM | MED-U | 2 | 28 | 2 | 1 | 1 | 8 | 1 | 1 | 1 | 1 | 0 | 1 | 34 | 29 | 1 | 3 | 1 | 1 | 20 | 0 | 0 | 0 | 136 | 2 |
| 12_YP54_IM | MED-U | 1 | 28 | 2 | 1 | 1 | 8 | 1 | 1 | 1 | 1 | 0 | 1 | 34 | 29 | 1 | 3 | 1 | 1 | 20 | 0 | 0 | 0 | 135 | 2 |
| 13_YP56_IM | MED-U | 2 | 28 | 2 | 1 | 1 | 8 | 1 | 1 | 1 | 1 | 0 | 1 | 34 | 29 | 1 | 3 | 1 | 1 | 20 | 0 | 0 | 0 | 136 | 2 |
| 14_YP75_IM | MED-U | 2 | 28 | 2 | 1 | 1 | 8 | 1 | 1 | 1 | 1 | 0 | 1 | 33 | 30 | 1 | 3 | 1 | 1 | 20 | 0 | 0 | 0 | 136 | 3 |
| 15_YP61_IM | MED-U | 1 | 28 | 2 | 1 | 1 | 8 | 1 | 1 | 1 | 1 | 0 | 1 | 34 | 29 | 1 | 3 | 1 | 1 | 20 | 0 | 0 | 0 | 135 | 2 |
| 16_YP53_IM | MED-U | 1 | 28 | 2 | 1 | 1 | 8 | 1 | 1 | 1 | 1 | 0 | 1 | 34 | 29 | 1 | 3 | 1 | 1 | 20 | 0 | 0 | 0 | 135 | 2 |
| 17_YP69_IM | MED-U | 2 | 28 | 2 | 1 | 1 | 8 | 1 | 1 | 1 | 1 | 0 | 1 | 35 | 28 | 1 | 3 | 1 | 1 | 20 | 0 | 0 | 0 | 136 | 2 |
| 18_YP64_IM | MED-U | 2 | 28 | 2 | 1 | 1 | 8 | 1 | 1 | 1 | 1 | 0 | 1 | 33 | 30 | 1 | 3 | 1 | 1 | 20 | 0 | 0 | 0 | 136 | 1 |
| 20_YP71_IM | MED-U | 2 | 28 | 2 | 1 | 1 | 8 | 1 | 1 | 1 | 1 | 0 | 1 | 34 | 29 | 1 | 3 | 1 | 1 | 20 | 0 | 0 | 0 | 136 | 2 |
| 21_YP63_IM | MED-U | 1 | 28 | 2 | 1 | 1 | 8 | 1 | 1 | 1 | 1 | 0 | 1 | 35 | 28 | 1 | 3 | 1 | 1 | 20 | 0 | 0 | 0 | 135 | 2 |
| 22_YP66_IM | MED-U | 1 | 28 | 2 | 1 | 1 | 8 | 1 | 1 | 1 | 1 | 0 | 1 | 33 | 30 | 1 | 3 | 1 | 1 | 20 | 0 | 0 | 0 | 135 | 2 |
| 23_YP72_IM | MED-U | 2 | 28 | 2 | 1 | 1 | 8 | 1 | 1 | 1 | 1 | 0 | 1 | 33 | 30 | 1 | 3 | 1 | 1 | 20 | 0 | 0 | 0 | 136 | 2 |
| 24_YP70_IM | MED-U | 2 | 28 | 2 | 1 | 1 | 8 | 1 | 1 | 1 | 1 | 0 | 1 | 34 | 29 | 1 | 3 | 1 | 1 | 20 | 0 | 0 | 0 | 136 | 2 |
| 25_YP73_IM | MED-U | 2 | 28 | 2 | 1 | 1 | 8 | 1 | 1 | 1 | 1 | 0 | 1 | 34 | 29 | 1 | 3 | 1 | 1 | 20 | 0 | 0 | 0 | 136 | 3 |
| 4_YP65_PKK | MED-U | 2 | 28 | 2 | 1 | 1 | 8 | 1 | 1 | 1 | 1 | 0 | 1 | 32 | 31 | 1 | 3 | 1 | 1 | 20 | 0 | 0 | 0 | 136 | 2 |
| 40_YP4_PB | MED-U | 0 | 28 | 2 | 1 | 1 | 8 | 1 | 1 | 1 | 1 | 0 | 1 | 31 | 32 | 1 | 3 | 1 | 1 | 20 | 0 | 0 | 0 | 134 | 2 |
| 41_YP26_PAK | MED-U | 0 | 28 | 2 | 1 | 1 | 8 | 1 | 1 | 1 | 1 | 0 | 1 | 35 | 28 | 1 | 3 | 1 | 1 | 20 | 0 | 0 | 0 | 134 | 2 |
| 43_YP6_UE | MED-U | 1 | 28 | 2 | 1 | 1 | 8 | 1 | 1 | 1 | 1 | 0 | 1 | 34 | 29 | 1 | 3 | 1 | 1 | 20 | 0 | 0 | 0 | 135 | 2 |
| 44_YP7_NP | MED-U | 1 | 28 | 2 | 1 | 1 | 8 | 1 | 1 | 1 | 1 | 0 | 1 | 34 | 29 | 1 | 3 | 1 | 1 | 20 | 0 | 0 | 0 | 135 | 2 |
| 45_YP8_PAK | MED-U | 1 | 28 | 2 | 1 | 1 | 8 | 1 | 1 | 2 | 0 | 0 | 1 | 34 | 29 | 1 | 3 | 1 | 1 | 20 | 0 | 0 | 0 | 135 | 2 |
| 46_YP29_PAK | MED-U | 2 | 28 | 2 | 1 | 1 | 8 | 1 | 1 | 1 | 1 | 0 | 1 | 34 | 29 | 1 | 3 | 1 | 1 | 20 | 0 | 0 | 0 | 136 | 2 |
| 47_YP36_PAK | MED-U | 2 | 28 | 2 | 1 | 1 | 8 | 1 | 1 | 1 | 1 | 0 | 1 | 34 | 29 | 1 | 3 | 1 | 1 | 20 | 0 | 0 | 0 | 136 | 2 |
| 48_YP14_PAK | MED-U | 0 | 44 | 1 | 1 | 1 | 9 | 1 | 1 | 2 | 0 | 0 | 1 | 31 | 26 | 1 | 3 | 1 | 1 | 20 | 0 | 0 | 0 | 144 | 3 |
| 50_YP23_PB | MED-U | 0 | 28 | 1 | 1 | 1 | 8 | 1 | 1 | 1 | 1 | 0 | 1 | 33 | 30 | 1 | 3 | 1 | 1 | 21 | 0 | 0 | 0 | 134 | 2 |
| 51_YP1_PAK | MED-U | 0 | 28 | 2 | 1 | 1 | 8 | 1 | 1 | 1 | 1 | 0 | 1 | 36 | 27 | 1 | 3 | 1 | 1 | 20 | 0 | 0 | 0 | 134 | 2 |
| 52_YP18_PAK | MED-U | 0 | 28 | 2 | 1 | 1 | 8 | 1 | 1 | 1 | 1 | 0 | 1 | 34 | 29 | 1 | 3 | 1 | 1 | 20 | 0 | 0 | 0 | 134 | 2 |
| 56_YP12_TK | MED-U | 0 | 28 | 2 | 1 | 1 | 8 | 1 | 1 | 1 | 1 | 0 | 1 | 34 | 29 | 1 | 3 | 1 | 1 | 20 | 0 | 0 | 0 | 134 | 2 |
| 58_YP9_TK | MED-U | 0 | 28 | 1 | 1 | 1 | 8 | 1 | 1 | 1 | 1 | 0 | 1 | 32 | 31 | 1 | 3 | 1 | 1 | 21 | 0 | 0 | 0 | 134 | 2 |
| 59_YP21_IM | MED-U | 1 | 28 | 1 | 1 | 1 | 8 | 1 | 1 | 1 | 1 | 0 | 1 | 32 | 31 | 1 | 3 | 1 | 1 | 21 | 0 | 0 | 0 | 135 | 2 |
| 60_YP24_IM | MED-U | 1 | 28 | 2 | 1 | 1 | 8 | 1 | 1 | 1 | 1 | 0 | 1 | 36 | 27 | 1 | 3 | 1 | 1 | 20 | 0 | 0 | 0 | 135 | 2 |
| 61_YP3_PB | MED-U | 1 | 28 | 2 | 1 | 1 | 8 | 1 | 1 | 1 | 1 | 0 | 1 | 33 | 30 | 1 | 3 | 1 | 1 | 20 | 0 | 0 | 0 | 135 | 2 |
| 62_YP33_PB | MED-U | 1 | 28 | 2 | 1 | 1 | 8 | 1 | 1 | 1 | 1 | 0 | 1 | 34 | 29 | 1 | 3 | 1 | 1 | 20 | 0 | 0 | 0 | 135 | 2 |
| 63_YP2_PB | MED-U | 2 | 28 | 2 | 1 | 1 | 8 | 1 | 1 | 1 | 1 | 0 | 1 | 35 | 28 | 1 | 3 | 1 | 1 | 20 | 0 | 0 | 0 | 136 | 2 |
| 66_YP38_PB | MED-U | 2 | 28 | 2 | 1 | 1 | 8 | 1 | 1 | 1 | 1 | 0 | 1 | 34 | 29 | 1 | 3 | 1 | 1 | 20 | 0 | 0 | 0 | 136 | 2 |
| 67_YP34_PB | MED-U | 1 | 28 | 2 | 1 | 1 | 8 | 1 | 1 | 2 | 0 | 0 | 1 | 34 | 29 | 1 | 3 | 1 | 1 | 20 | 0 | 0 | 0 | 135 | 2 |
| 7_YP60_IM | MED-U | 1 | 28 | 2 | 1 | 1 | 8 | 1 | 1 | 1 | 1 | 0 | 1 | 35 | 28 | 1 | 3 | 1 | 1 | 20 | 0 | 0 | 0 | 135 | 2 |
| 70_YP27_IM | MED-U | 0 | 28 | 2 | 1 | 1 | 8 | 1 | 1 | 1 | 1 | 0 | 1 | 34 | 29 | 1 | 3 | 1 | 1 | 20 | 0 | 0 | 0 | 134 | 2 |
| 71_YP7_IM | MED-U | 0 | 28 | 2 | 1 | 1 | 8 | 1 | 1 | 1 | 1 | 0 | 1 | 34 | 29 | 1 | 3 | 1 | 1 | 20 | 0 | 0 | 0 | 134 | 2 |
| 73_YP21_KK | MED-U | 2 | 28 | 2 | 1 | 1 | 8 | 1 | 1 | 1 | 1 | 0 | 1 | 34 | 29 | 1 | 3 | 1 | 1 | 20 | 0 | 0 | 0 | 136 | 3 |
| 75_YP26_PB | MED-U | 0 | 28 | 1 | 1 | 1 | 8 | 1 | 1 | 1 | 1 | 0 | 1 | 33 | 30 | 1 | 3 | 1 | 1 | 21 | 0 | 0 | 0 | 134 | 3 |
| 77_YP25_IM | MED-U | 1 | 28 | 2 | 1 | 1 | 8 | 1 | 1 | 1 | 1 | 0 | 1 | 35 | 28 | 1 | 3 | 1 | 1 | 20 | 0 | 0 | 0 | 135 | 2 |
| 81_YP39_IM | MED-U | 2 | 28 | 2 | 1 | 1 | 8 | 1 | 1 | 1 | 1 | 0 | 1 | 34 | 29 | 1 | 3 | 1 | 1 | 20 | 0 | 0 | 0 | 136 | 2 |
| 82_YP1_IM | MED-U | 1 | 28 | 2 | 1 | 1 | 8 | 1 | 1 | 2 | 0 | 0 | 1 | 34 | 29 | 1 | 3 | 1 | 1 | 20 | 0 | 0 | 0 | 135 | 2 |
| 83_YP13_ADT | MED-U | 2 | 28 | 2 | 1 | 1 | 8 | 1 | 1 | 1 | 1 | 0 | 1 | 34 | 29 | 1 | 3 | 1 | 1 | 20 | 0 | 0 | 0 | 136 | 2 |
| 9_YP68_IM | MED-U | 1 | 28 | 2 | 1 | 1 | 8 | 1 | 1 | 1 | 1 | 0 | 1 | 34 | 29 | 1 | 3 | 1 | 1 | 20 | 0 | 0 | 0 | 135 | 1 |
| 93_YP24_KK | MED-U | 2 | 28 | 2 | 1 | 1 | 8 | 1 | 1 | 1 | 1 | 0 | 1 | 32 | 31 | 1 | 3 | 1 | 1 | 20 | 0 | 0 | 0 | 136 | 2 |
| 35_YP14_TLH | Talas | 0 | 28 | 1 | 1 | 1 | 8 | 1 | 1 | 1 | 1 | 1 | 1 | 34 | 29 | 1 | 3 | 1 | 1 | 21 | 0 | 0 | 0 | 135 | 2 |
| 37_YP35_TLH | Talas | 0 | 28 | 1 | 1 | 1 | 8 | 1 | 1 | 1 | 1 | 1 | 1 | 34 | 29 | 1 | 3 | 1 | 1 | 21 | 0 | 0 | 0 | 135 | 2 |
| 39_YP13_TLH | Talas | 1 | 28 | 1 | 1 | 1 | 8 | 1 | 1 | 1 | 1 | 1 | 1 | 33 | 30 | 1 | 3 | 1 | 1 | 22 | 0 | 0 | 0 | 137 | 2 |
| 19_YP74_IM | UN | 0 | 5 | 1 | 0 | 2 | 3 | 1 | 3 | 1 | 1 | 0 | 1 | 4 | 1 | 0 | 3 | 1 | 1 | 7 | 1 | 2 | 0 | 38 | 4 |
| 36_YP27_TLH | UN | 0 | 5 | 1 | 0 | 1 | 3 | 1 | 3 | 1 | 1 | 0 | 1 | 4 | 1 | 0 | 3 | 1 | 1 | 7 | 1 | 2 | 0 | 37 | 3 |
| 49_YP5_PAK | UN | 1 | 28 | 2 | 1 | 1 | 8 | 1 | 1 | 1 | 1 | 0 | 1 | 33 | 30 | 1 | 3 | 1 | 1 | 20 | 0 | 0 | 0 | 135 | 2 |
| 57_YP22_IM | UN | 0 | 5 | 1 | 0 | 2 | 3 | 1 | 3 | 1 | 1 | 0 | 1 | 4 | 1 | 0 | 3 | 1 | 1 | 7 | 1 | 2 | 0 | 38 | 2 |

Abbreviations of clade names are: MED-D – Medievalis desert; MED-U – Medievalis upland; ANT – Antique; Talas – high-mountains Talas plague focus; YPT – *Y. pseudotuberculosis*; UN – unidentified.
