# Supplementary material for: Whole genome sequencing of Yersinia pestis isolates from Central Asian natural plague foci revealed the role of adaptation to different hosts and environmental conditions in shaping specific genotypes
Source: PLoS Negl Trop Dis. 2025 Sep 12;19(9):e0013533. doi: 10.1371/journal.pntd.0013533 (PMC12445494; doi:10.1371/journal.pntd.0013533)
Supplement: Attachment — Submitted filename: Plos Neglected Yersinia pestis.docx [file pntd.0013533.s011.docx]

Following a thorough analysis of the manuscript titled ***Whole genome sequencing of Yersinia pestis isolates from Central Asian natural plague foci revealed the role of adaptation to different hosts and environmental conditions in shaping specific genotypes*** , the reviewer would like to highlight several key issues that require particular attention.

1. The bacterium is referred to inconsistently throughout the manuscript (e.g., *Yersinia*, *Yersinia pestis*, ANT, MED, etc.), and biovar abbreviations are used in a non-standardized manner, which affects clarity and readability.
2. Several figure legends and descriptions lack complete information, making it difficult to interpret the presented data.
3. There is a noticeable mixing of results and discussion in some sections, which impacts the structural coherence of the manuscript.
4. The discussion section includes few references, weakening the support for some of the interpretations and conclusions.

Below are detailed comments and questions regarding the conducted research.

Lane 67-69: „The extensive distribution of these natural plague foci underscores the importance of continuous surveillance and targeted control measures to mitigate the risk of human plague outbreaks in Kazakhstan [16].” Which ones? Add some exemples for control measures and action.

Lane 72-73: „Plague outbreaks can occur even in industrially advanced countries, as evidenced by the outbreak in Colorado (USA) during June-July 2014 [20].” Give some numbers from Colorado

Lanes: 82-113 Please explain or provide some reasons why biochemical traits should be replaced or supported by other modern techniques. Indicate which methods currently represent the main techniques used for identification.

Lane: 82-85: Please also include references here, prior to the detailed information provided below.

Lane 81. Please add a dot at the end of the sentence.

Lane 131: Yersinia pestis strains used in this study. Name of bacteria in *italics* (in the subchapter title). Please provide in the main text the range of years during which this bacterium was isolated. Also give the numbers of strains from particular source tested within this work, for example, how many strains from Great gerbil (n=33?) and so on.

Upon a thorough review of the strain list in Supplementary Table 1, questions arise regarding whether the selection of strains for this study ensures the formulation of reliable conclusions. Specifically, there is an imbalance in the representation of strains from different regions and host species. I understand that fulfilling such a condition might be difficult due to logistical and ecological constraints, but how would you address a potential concern from a microbiological perspective regarding the generalizability and robustness of the findings, given this sampling limitation? Furthermore, what criteria did you use when selecting strains from the collection?

Lane 148. There should be a dot at the end of the sentence.

Lane 150: Fermentation and denitrification subchapter – Please provide the necessary references for the methodology.

Table 1: Please correct the table, as all primers have identical names. Expand the primer names and clearly indicate which ones correspond to specific biovars. Additionally, include the expected amplicon sizes for each primer pair. Remember to include details about longer PCR amplicons with these primers that distinguish them from the characteristic amplicons for MED isolates. This information is currently missing in the method description below.

Kindly ask to move this information from Supplemental Table S2. Furthermore, it is unclear in Supplemental Table S2 which primers correspond to which biovars. I suggest adding a "+" or "-" to indicate the biovars, or alternatively, including this information in Table 1 in the main text.

Lane 221-222: Y. pestis ANT or ANT biovar. Please unify.

Lane 224: Abbreviations such as Medieval (MED) biovar, etc., should be explained earlier and used consistently throughout the entire work.

Lane 281: „Y. pestis bv. Orientalis” please unify the names of strains/isolates. It can be see few types of description, please unify. What is more, Lanes 281-283 looks like disscusion.

**Please review the entire manuscript carefully for inconsistent strain nomenclature. It is essential to standardize the naming format and apply a uniform style consistently throughout the work.**

Lane 290-291: „It can therefore be concluded that the Talas isolates might be closer to the Altay strains, which is unexpected, as the Gissar Range is geographically closer.” Another example of discussion is provided here, which is understandable; however, it is misplaced within this section. The current structure introduces a degree of confusion. I suggest reconsidering the organization of the entire section titled “Y. pestis biovar identification and typing using whole genome sequences” and potentially restructuring it as part of the Discussion section instead. This would improve the logical flow and contextual coherence of the manuscript.

Lane 329: „The length of the sequence between the Med24 primers (Table 1) varied among different isolates. ” In this table, there is a lack of information about the length. See comment above. It is worth considering making changes.

Lane 333: In all other strains, the sequence was shorter (207 bp) due to a deletion. Could you indicate where this result is presented? Please provide an appropriate reference or point to the relevant figure or table.

Lane 352: Give a legend and mark which reference strain are for Y. pestis / Y. pseudotuberculosis.

Lane 394-396: „Furthermore, assuming that genetic polymorphisms are driven by selective pressures from adaptation to specific hosts or environmental conditions, it cannot be ruled out that distinct bacteria might develop similar patterns of polymorphic sites through convergent evolution.” Could you provide a reference to support this claim, particularly in relation to Y. pestis strains from regions outside the study area?

Lane 402-405: How do you explain the presence of desert-origin strains in the upland branch and upland-origin strains in the desert branch? Please elaborate on this phenomenon considering the specific characteristics associated with these strains.

Lane 474-476: „Strain 53_YP3_IM19 was found to lack any plasmids. An analysis of SPAdes-assembled contigs for this strain did not reveal any contigs that could be associated with known or unknown plasmids.” Is there any explanation for this observation? Have you discussed this result in the manuscript?

Lane 477-480: Are you sure this paragraph is best placed here? Wouldn’t it be more appropriate in the Introduction or Discussion section?

Lane 558 (Y. pestis strains identified as the MED biovar) or Lane 563: In general, Yersinia strains. Another example of inconsistent naming.

Lane 558: The statement that 'Y. pestis strains identified as the MED biovar were the most abundant among Yersinia isolates from Central Asia and the Caucasus, and that these strains were strongly associated with gerbil populations, their fleas, and ticks, which may play a role in the distribution of the infection,' appears to reference established knowledge from existing scientific sources. If this is your original conclusion, could you please provide further clarification? Specifically, considering that the number of strains derived from different animal or sources is not similar in your study, as well as the fact that the strains were selected from a collection rather than isolated directly from animals/sources, how confident are you in asserting such a direct association? I would appreciate a more detailed justification for this claim or supporting references, especially considering that the set of strains was selected rather than newly isolated.

Lane 581: Please provide a legend for the figure, or indicate which branch is represented by each species. Additionally, include more information about the strains in this figure. Your description: „Figure 2. Phylogenetic tree designed by the progressiveMauve alignment of chromosomal sequences of several selected Yersinia isolates and reference genomes” is not sufficiently informative.

Lack of many references in the discussion part. Section results are mixed with disccusion phrases.

**Supplemental Table S1. *Yersinia pestis* strains used in this study. There is no information provided regarding the biovar classification. A possible solution would be to create a more comprehensive, yet consolidated, table by combining selected data from multiple existing tables. The current distribution across numerous supplementary tables makes it inconvenient to access key information at different stages of the manuscript. I recommend revising the layout by either merging, clarifying, or reducing the number of tables to improve readability and usability.**

Supplemental Table S3. Give legend for table. As you gave for Table S5: Abbreviations of clade names are: MED-D – Medievalis desert; MED-U – Medievalis upland; ANT – Antique; Talas – high-mountains Talas plague focus; YPT – *Y. pseudotuberculosis*; UN – unidentified.
