# Supplementary material for: Whole genome sequencing of Yersinia pestis isolates from Central Asian natural plague foci revealed the role of adaptation to different hosts and environmental conditions in shaping specific genotypes
Source: PLoS Negl Trop Dis. 2025 Sep 12;19(9):e0013533. doi: 10.1371/journal.pntd.0013533 (PMC12445494; doi:10.1371/journal.pntd.0013533)
Supplement: Attachment — Submitted filename: point-to-point answers_17.07.2025.docx [file pntd.0013533.s013.docx]

# Reviewer #1

Following a thorough analysis of the manuscript titled ***Whole genome sequencing of Yersinia pestis isolates from Central Asian natural plague foci revealed the role of adaptation to different hosts and environmental conditions in shaping specific genotypes*** , the reviewer would like to highlight several key issues that require particular attention.

1. The bacterium is referred to inconsistently throughout the manuscript (e.g., *Yersinia*, *Yersinia pestis*, ANT, MED, etc.), and biovar abbreviations are used in a non-standardized manner, which affects clarity and readability.
2. Several figure legends and descriptions lack complete information, making it difficult to interpret the presented data.
3. There is a noticeable mixing of results and discussion in some sections, which impacts the structural coherence of the manuscript.
4. The discussion section includes few references, weakening the support for some of the interpretations and conclusions.

Below are detailed comments and questions regarding the conducted research.

Dear reviewer,

Thank you very much for your thoughtful recommendations. Below is a point-by-point list of the corrections we have implemented to address your comments.

Lane 67-69: „The extensive distribution of these natural plague foci underscores the importance of continuous surveillance and targeted control measures to mitigate the risk of human plague outbreaks in Kazakhstan [16].” Which ones? Add some exemples for control measures and action.

This statement was added (lines 102-104): „These measures include regular sample collection for pathogen monitoring by specialized anti-plague stations, vaccination of at-risk populations and camels, disinfection and deratization of villages and livestock shelters, and public education efforts [23].”

Lane 72-73: „Plague outbreaks can occur even in industrially advanced countries, as evidenced by the outbreak in Colorado (USA) during June-July 2014 [20].” Give some numbers from Colorado

Text was modified in lines 107-110: „Plague outbreaks can occur even in industrially advanced countries, as evidenced by the outbreak in Colorado (USA) during June-July 2014 [27], when four separate human plague infections were diagnosed. These included the owners of an infected dog and veterinary workers, all of whom became infected after exposure to the sick animal.”

Lanes: 82-113 Please explain or provide some reasons why biochemical traits should be replaced or supported by other modern techniques. Indicate which methods currently represent the main techniques used for identification.

This paragraph with two new references was added in lines 151-153:
„While biochemical tests and PCR-based VNTR/MLVA typing are widely used for the characterization of *Y. pestis* isolates [38, 39], these approaches are sometimes inconclusive for atypical strains and do not provide sufficient resolution for monitoring individual clonal lineages of the pathogen in nature.”

It is important to note that we are not suggesting a replacement of traditional biochemical tests with modern techniques.

Lane: 82-85: Please also include references here, prior to the detailed information provided below.

A reference to Zhou et al (2004) was added.

Lane 81. Please add a dot at the end of the sentence.

Fixed.

Lane 131: Yersinia pestis strains used in this study. Name of bacteria in *italics* (in the subchapter title).

Please note that when the main title text is in italic font, Latin species names should be written in plain (non-italic) text. It can be changed by the production editor at the end.

Please provide in the main text the range of years during which this bacterium was isolated.

The following text was added in lines 184-188: „Strains were isolated from different regions during field work expeditions for sample collection from natural plague foci in Central Asia over a long period, starting in 1961 (the earliest strain) and continuing through to recent years, with the latest strain collected in 2022 (see Suppl. Table S1). The strains were selected to include both typical and several atypical isolates representing various desert and mountain plague foci of Central Asia.”

Also give the numbers of strains from particular source tested within this work, for example, how many strains from Great gerbil (n=33?) and so on.

This information is given in Suppl. Table S1. If you think that this information if of significant importance, the table can be moded from supplementary materials to the main text.

A more detailed breakdown of the strain origin cannot be easily implemented due to the fact that each strain is characterized by multiple variables: geographic origin, host or carrier animal, and landscape. A three-dimensional table would be required to present the data properly. All this information is already provided in flat supplementary tables. However, we agree that the number of strains per category is important for the statistical validation of the results. This issue is addressed in the next question.

Upon a thorough review of the strain list in Supplementary Table 1, questions arise regarding whether the selection of strains for this study ensures the formulation of reliable conclusions. Specifically, there is an imbalance in the representation of strains from different regions and host species. I understand that fulfilling such a condition might be difficult due to logistical and ecological constraints, but how would you address a potential concern from a microbiological perspective regarding the generalizability and robustness of the findings, given this sampling limitation? Furthermore, what criteria did you use when selecting strains from the collection?

Please consider that one of the main conclusions of the paper is that MED isolates from desert areas can be genetically distinguished from upland isolates. The number of strains in these two branches of the dendrogram in Figure 3 (former Figure 4) is comparable. It would be quite difficult to design a collection of isolates in which strains from hosts and insect vectors, from different host species, and from different geographical regions are equally represented across all possible dimensions.

In the current version of the manuscript, an additional statistical analysis was included to validate the significance of the separation between the desert and upland variants of the MED isolates. A new subsection „Statistical validation of clade segregation by genetic polymorphisms” was added to the Methods (lines 296-304).

This text added to the Results (lines 472-476): „The significance of segregation between desert and upland variants of *Y. pestis* MED genomes was validated using a random forest classifier, which achieved a classification accuracy of 0.9889. This indicates that the likelihood of misclassifying isolates from different environments based on the selected genetic polymorphisms is approximately 1%. A bootstrap analysis of clustering robustness confirmed the separation of strains with a p-value of 0.0.”

Lane 148. There should be a dot at the end of the sentence.

Fixed

Lane 150: Fermentation and denitrification subchapter – Please provide the necessary references for the methodology.

Text was added (line 210): „Tests used in this study were described in publication by Eroshenko et al. (2023) [44].”

Table 1: Please correct the table, as all primers have identical names.

Column heading was changed from „Primer names” to „Primer directions”.

Expand the primer names and clearly indicate which ones correspond to specific biovars. Additionally, include the expected amplicon sizes for each primer pair. Remember to include details about longer PCR amplicons with these primers that distinguish them from the characteristic amplicons for MED isolates. This information is currently missing in the method description below.

A new column „Expected PCR products” was added to Table 1.

Kindly ask to move this information from Supplemental Table S2. Furthermore, it is unclear in Supplemental Table S2 which primers correspond to which biovars. I suggest adding a "+" or "-" to indicate the biovars, or alternatively, including this information in Table 1 in the main text.

Biovar identification is provided in Suppl. Table S3. The issuer of taxonomic labeling of the isolates will also be discussed in detail below , in response to another question.

Lane 221-222: Y. pestis ANT or ANT biovar. Please unify.

There was an error. The text has been revised as follows (lines 284-286): „The genome of *Y. pestis* strain SCPM-O-B-6899 (CP045145) was used as the reference. This strain was selected because it is equidistant from all current isolates and possesses the rare plasmid pCKF [14, 15].”

Lane 224: Abbreviations such as Medieval (MED) biovar, etc., should be explained earlier and used consistently throughout the entire work.

The abbreviations MED and ANT were introduced in the Introduction (lines 68-69). The word 'Medieval' was removed from line 224 and replaced with the abbreviation MED.

Lane 281: „Y. pestis bv. Orientalis” please unify the names of strains/isolates. It can be see few types of description, please unify. What is more, Lanes 281-283 looks like disscusion.

Text of this paragraph was modified (lines 348-350): „The biovar ORI can be distinguished from other biovars by a 93 bp deletion in aerobic glycerol-3-phosphate dehydrogenase *glpD* [11, 35]. None of the isolates had this deletion that was in good correspondence with the actual glycerine fermentation test.”

**Please review the entire manuscript carefully for inconsistent strain nomenclature. It is essential to standardize the naming format and apply a uniform style consistently throughout the work.**

Thank you for this recommendation. The text of the paper has been thoroughly checked.

The strain collection selected for this study includes several isolates initially identified phenotypically as *Y. pestis*; however, genomic analysis revealed their similarity to *Y. pseudotuberculosis*. Therefore, it would not be accurate to refer to all strains as *Y. pestis* isolates, and we used *Yersinia* when referring to the entire collection.

The abbreviations ANT and MED for the Antiqua and Medievalis biovars are used in other publications. They are introduced in lines 68-69 of the Introduction. If the reviewer can suggest more standard abbreviations for these biovars, we will gladly consider adopting them.

Lane 290-291: „It can therefore be concluded that the Talas isolates might be closer to the Altay strains, which is unexpected, as the Gissar Range is geographically closer.” Another example of discussion is provided here, which is understandable; however, it is misplaced within this section. The current structure introduces a degree of confusion. I suggest reconsidering the organization of the entire section titled “Y. pestis biovar identification and typing using whole genome sequences” and potentially restructuring it as part of the Discussion section instead. This would improve the logical flow and contextual coherence of the manuscript.

This speculative discussion has been replaced with this neutral phrase (lines 354-357): „According to Kislichkina et al. (2015), the Gissar isolates could be distinguished from the sister Altay strains by a G→A substitution at the 482^nd^ nucleotide position in the rhamnose operon transcriptional activator *rhaS* [54]. This substitution has not been observed in the analyzed strains.”

Lane 329: „The length of the sequence between the Med24 primers (Table 1) varied among different isolates. ” In this table, there is a lack of information about the length. See comment above. It is worth considering making changes.

The required information was added to Table 1.

Lane 333: In all other strains, the sequence was shorter (207 bp) due to a deletion. Could you indicate where this result is presented? Please provide an appropriate reference or point to the relevant figure or table.

A reference to Suppl. Table S3 was added.

Lane 352: Give a legend and mark which reference strain are for Y. pestis / Y. pseudotuberculosis.

The figure legend was modified:
Figure 2. Phylogenetic tree designed by the progressiveMauve alignment of chromosomal sequences of several selected *Yersinia* isolates and the reference genomes *Y. pestis* SCPM-O-B-6899 and *Y. pseudotuberculosis* IP 32953.

Lane 394-396: „Furthermore, assuming that genetic polymorphisms are driven by selective pressures from adaptation to specific hosts or environmental conditions, it cannot be ruled out that distinct bacteria might develop similar patterns of polymorphic sites through convergent evolution.” Could you provide a reference to support this claim, particularly in relation to Y. pestis strains from regions outside the study area?

This phrase was added (lines 461-463): „For example, in a paper by Mas Fiol et al. (2024), it was reported that approximately 36% of genes in sequenced *Y. pestis* genomes harbored convergent (homoplastic) nonsynonymous mutations [58].”

Lane 402-405: How do you explain the presence of desert-origin strains in the upland branch and upland-origin strains in the desert branch? Please elaborate on this phenomenon considering the specific characteristics associated with these strains.

It was explained here (lines 470-471): „... some level of introgression between the regions, particularly from the uplands toward the surrounding deserts.”

This concept was more elaborated in the discussion (lines 629-637):
„This analysis revealed a clear separation of the MED strains into two branches, primarily isolated from desert and upland regions of Central Asia. In general, *Y. pestis* strains isolated from uplands and mountain valleys were genetically more diverse than those from the desert plains of Central Asia. This can be attributed to the isolation of host populations in mountainous regions. Overpopulation of rodents in confined mountain valleys likely drives animals to migrate toward surrounding deserts, creating a flow of migrants from the mountains to the desert regions. This continuous movement may limit the influx of bacterial pathogen variants from external sources. This assumption is supported by the higher observed frequency of introgression of upland variants of *Y. pestis* into desert areas, compared to the reverse (Figure 3).”

We cannot elaborate it more without running into speculations.

Lane 474-476: „Strain 53_YP3_IM19 was found to lack any plasmids. An analysis of SPAdes-assembled contigs for this strain did not reveal any contigs that could be associated with known or unknown plasmids.” Is there any explanation for this observation? Have you discussed this result in the manuscript?

Strain 53_YP3_IM19 is rather unique in all aspects. It shows clear similarity to *Y. pseudotuberculosis* and may represent an upland sublineage of this species that was erroneously identified as *Y. pestis*. This organism is not the focus of the current study but was included to highlight the limitations of traditional bacteriological methods for species identification based on phenotype. We cannot provide further insight into the biological reasons for the absence of plasmids, whether they were lost or never existed in this lineage remains unclear.

Lane 477-480: Are you sure this paragraph is best placed here? Wouldn’t it be more appropriate in the Introduction or Discussion section?

This paragraph was moved to the Introduction. An additional text was added regarding the role of plasmid in *Y. pestis* virulence. See lines 58-71.

Lane 558 (Y. pestis strains identified as the MED biovar) or Lane 563: In general, Yersinia strains. Another example of inconsistent naming.

The species naming was checked thoughout the text.

Lane 558: The statement that 'Y. pestis strains identified as the MED biovar were the most abundant among Yersinia isolates from Central Asia and the Caucasus, and that these strains were strongly associated with gerbil populations, their fleas, and ticks, which may play a role in the distribution of the infection,' appears to reference established knowledge from existing scientific sources. If this is your original conclusion, could you please provide further clarification? Specifically, considering that the number of strains derived from different animal or sources is not similar in your study, as well as the fact that the strains were selected from a collection rather than isolated directly from animals/sources, how confident are you in asserting such a direct association? I would appreciate a more detailed justification for this claim or supporting references, especially considering that the set of strains was selected rather than newly isolated.

The fact that *Y. pestis* MED isolates are predominantly associated with gerbils and their fleas, whereas ANT isolates are associated with marmots, is not new. We have added three references [23, 67, 68] to support this statement. Since this association is already well established, we did not focus our study on confirming these pathogen-host relationships. Conversely, the finding that MED strains from desert and mountain landscapes are distinguishable by genotype is entirely novel.

Additionally, we confirm that the collection strains used in this study are properly documented with respect to their sources of isolation.

Lane 581: Please provide a legend for the figure, or indicate which branch is represented by each species. Additionally, include more information about the strains in this figure. Your description: „Figure 2. Phylogenetic tree designed by the progressiveMauve alignment of chromosomal sequences of several selected Yersinia isolates and reference genomes” is not sufficiently informative.

Figure 2 legend was modified as it was explained above.

Lack of many references in the discussion part. Section results are mixed with disccusion phrases.

More references were added.

**Supplemental Table S1. *Yersinia pestis* strains used in this study. There is no information provided regarding the biovar classification. A possible solution would be to create a more comprehensive, yet consolidated, table by combining selected data from multiple existing tables. The current distribution across numerous supplementary tables makes it inconvenient to access key information at different stages of the manuscript. I recommend revising the layout by either merging, clarifying, or reducing the number of tables to improve readability and usability.**

Supplemental Table S3. Give legend for table. As you gave for Table S5: Abbreviations of clade names are: MED-D – Medievalis desert; MED-U – Medievalis upland; ANT – Antique; Talas – high-mountains Talas plague focus; YPT – *Y. pseudotuberculosis*; UN – unidentified.

Dear reviewer,

We are grateful for your thorough review of our manuscript and for your helpful comments and suggestions. We truly appreciate your time and effort.

We have done our best to facilitate navigation through the multiple supplementary tables referenced in this paper. In Suppl. Table 1, all isolates are arranged by plague foci, with the final letters in each strain name representing an abbreviated name of the corresponding focus.

In all other tables, strains are ordered by their assigned numbers.

Figure 4 summarizes the strain genotypes, geographic locations and host or carrier species.

There are several reasons why strain lineage classifications are not included in every table. First, to maintain a layout narrow enough to be easily readable on standard computer screens. Second, to follow the logical progression of the manuscript. We avoided labeling strains as MED-D and MED-U in tables referenced before this classification is formally introduced, as doing so could be misleading. This precaution is particularly important given that, due to introgressions, some strains genotyped as MED-U were isolated in desert regions, and vice versa.

The main aim of the paper is to demonstrate the genetic plasticity of the *Y. pestis* population, where traits inherited from common ancestors overlap with those shaped by environmental adaptation and horizontal gene transfer involving plasmids, transposons, and other mobile genetic elements. Premature or overly rigid labeling of strains early in the paper could distract readers from this central concept.

# Reviewer #2

**Are the objectives of the study clearly articulated with a clear, testable hypothesis stated?**
Partially.
The manuscript has well-described aims: to characterise Y. pestis isolates from Central Asia and identify genetic polymorphisms associated with ecological adaptation and lineage divergence. However, a clear hypothesis is not explicitly stated, and the narrative blends surveillance goals with exploratory phylogenomic analysis. For a stronger framing, the authors should articulate a central hypothesis (e.g., “Y. pestis genomic variation correlates with host or environmental origin more than with clonal ancestry”) early in the Introduction.

Thank you for this valuable suggestion. In response, we have added the following sentence at the end of the Introduction (lines 169-172) to clearly state the central hypothesis:

„The central hypothesis was that genetic variation in *Y. pestis* may correlate with host or environmental origin potentially obscuring clonal ancestry, thereby facilitating pathogen surveillance and offering new insights into its evolutionary dynamics.”

**Is the study design appropriate to address the stated objectives?**
Yes, broadly.
The use of WGS for 98 Y. pestis isolates, with phenotypic testing and in silico variant analysis, is appropriate for evaluating biovar classification, polymorphisms, and lineage diversity. However, comparative analyses with global strains are missing, which limits broader inference and validation of their findings.

We agree that including a comparative analysis with global reference strains such as KIM, Antiqua, Nepal515, and CO92 would be of interest. However, we ultimately decided against this approach because many of these strains were sequenced a long time ago using outdated technologies, such as Roche 454, which could raise concerns about data quality and compatibility for direct comparison with our data generated using the modern Illumina technology.

**Is the population clearly described and appropriate for the hypothesis being tested?**
Yes.
The manuscript provides detailed ecological and geographic context for the 98 isolates, spanning desert and upland plague foci. The inclusion of multiple hosts (e.g., gerbils, marmots, fleas) is appropriate given the zoonotic ecology of plague. A more structured breakdown of how many isolates came from which host species or region would help clarify population stratification.

Thank you for your supportive comment.

A more structured breakdown of the strain origins is challenging to implement because each isolate is defined by multiple variables including geographic origin, host or carrier species, and landscape type. Representing this information accurately would require a three-dimensional table. However, all of these details are provided in the flat-format supplementary tables.

The population-level statistical approaches we used to address stratification are explained in responses to other reviewer comments below.

**Is the sample size sufficient to ensure adequate power to address the hypothesis being tested?**
Generally yes, but with caveats.
For regional genomic surveillance, the sample size is robust (n = 98). However, comparisons across clades or ecological categories (e.g., desert vs upland) sometimes involve much smaller groups (e.g., 4 Talas isolates), which limits statistical power. The authors should acknowledge this limitation, particularly when making ecological inferences.

We acknowledge this important point. Due to the limitations noted, our conclusions focus specifically on the distinction between the "desert" and "upland" variants of the MED biovar, where the numbers of isolates are comparable and allow for more balanced comparison.

However, we agree that the initial statistical support for this conclusion was insufficient. To address this, we have included additional statistical validation, as described in response to the following question.

**Were correct statistical analyses used to support conclusions?**
Partially.
The authors use custom SNP calling, clustering, and dendrogram construction, but they rely heavily on parsimony-based clustering and descriptive comparisons without formal phylogenetic or population structure modelling. No statistical tests (e.g., Mantel tests, AMOVA, PCA) are used to quantify associations between genotype and geography/host. This weakens the analytical rigour and should be addressed with additional comparative or phylogenetic methods if claims about convergent evolution or ecological adaptation are to be substantiated.

We agree with this criticism and have addressed it by performing an additional statistical analysis, now described in a new subsection of the Methods section (lines 296-304):

*Statistical validation of clade segregation by genetic polymorphisms*

Clade segregation accuracy was assessed using the random forest classification algorithm, implemented via the RandomForestClassifier and model_selection.cross_val_score functions from the Python library sklearn (version 1.7.0). To evaluate whether the observed distribution of strains among clusters based on genetic polymorphism analysis significantly differs from a random distribution, a bootstrap analysis with 1,000 iterations was performed. In each iteration, the strains were randomly assigned to clusters, and classification accuracy scores were calculated. The likelihood that the accuracy from a random assignment equals or exceeds the actual score was estimated as a p-value using the mean function from the NumPy library (version 2.2.4).

The following results were added to the Results section (lines 472-476):

„The significance of segregation between desert and upland variants of Y. pestis MED genomes was validated using a random forest classifier, which achieved a classification accuracy of 0.9889. This indicates that the likelihood of misclassifying isolates from different environments based on the selected genetic polymorphisms is approximately 1%. A bootstrap analysis of clustering robustness confirmed the separation of strains with a p-value of 0.0.”

**Are there concerns about ethical or regulatory requirements being met?**
No major concerns raised, but additional clarity would help.
The authors state that isolates came from a national strain collection and were collected during routine surveillance. However: There is no explicit statement of ethical or biosafety approvals for working with Y. pestis (a BSL-3 agent).

We appreciate the reviewer’s attention to biosafety and ethical compliance. The following text was added at the beginning of the section Methods (lines 178-184): „All work involving *Yersinia pestis* was conducted in full accordance with national biosafety regulations and international standards for handling BSL-3 agents. Routine surveillance, strain maintenance, and research activities are carried out under institutional biosafety protocols approved by the Biosafety and Ethics Committee of the Institute. The use of *Y. pestis* strains from the NCMO for this study did not involve work with live animals or human subjects and was performed exclusively on archived bacterial cultures in compliance with national and institutional regulations.”

**Does the analysis presented match the analysis plan?**
Broadly yes, but exploratory in nature.
The study presents genome sequencing, variant detection, plasmid profiling, and phenotypic tests of Y. pestis isolates as planned. The described pipelines and laboratory methods align with the Results section. However, because the manuscript is exploratory rather than hypothesis-driven, there is no formal analysis plan or pre-specified comparisons. The selection of SNPs and construction of polymorphism matrices are well described, but the lack of formal statistical testing makes the structure more observational than analytical.

The questions regarding the work hypothesis and the statistical validation were answered above.

**Are the results clearly and completely presented?**
Partially.
The authors provide extensive detail, including strain-level variation, allele states, plasmid content, and CRISPR/transposon profiles. However, the narrative is dense, with long blocks of text that lack synthesis or summarisation.

Some key findings (e.g., upland vs desert divergence, pCKF identification) are buried in technical detail, making them hard to extract.

The lack of statistical tests or quantitative summaries (e.g., number of unique SNPs per clade, plasmid prevalence by ecotype) weakens the clarity of the results.

Recommendation: Add a concise summary table per major section (e.g., SNP patterns, plasmid presence, phenotypic test outcomes) and integrate clearer narrative transitions.

Thank you for these constructive suggestions. The Results and Discussion sections have been revised to improve clarity and include statistical validation of key findings. Additional references have also been incorporated to support the interpretations. We kindly ask you to review the updated sections to see whether the concerns have been adequately addressed. If any points remain unclear, we would appreciate more specific guidance on which paragraphs or statements require further elaboration.

Regarding the recommendation to add concise summary tables: our intention was to fulfill this purpose with the Figure 3 (previously Figure 4), which presents a structured summary of genotypes, ecotypes, and key features. This figure is supported by data presented across four supplementary tables.

**Are the figures (Tables, Images) of sufficient quality for clarity?**
No

Several figures are blurry or low-resolution, particularly Figures 2–5, which are central to the interpretation of genomic variation and lineage structure.

Font sizes are too small in dendrogram labels and SNP context annotations.

All figures were originally prepared and submitted as vector graphics in PDF format. The blurring likely resulted from the low-quality rendering during the manuscript PDF compilation process. We expect the image quality to be significantly improved in the final publication layout.

Figure 3 (pipeline diagram) is unnecessary and should be removed or moved to Supplementary Material.

The figure was moved to supplementary materials as recommended

Supplementary tables are useful but need better integration into the main narrative.

The supplementary tables include isolation source metadata, laboratory testing results, and the genotyping matrix. These datasets are discussed in detail throughout the manuscript, with explicit references directing readers to the relevant supplementary tables. This approach ensures that the supplementary materials are fully integrated into the main narrative and support the interpretation of the results. If the integration of supplementary tables into the text of manuscript can be improved, we would appreciate more specific guidance from the reviewer.

Reviewer #2: Are the conclusions supported by the data presented?
Partially.
The conclusions - particularly regarding the ecological structuring of Y. pestis genotypes (upland vs desert) and the possibility of convergent evolution - are intriguing and plausibly supported by the data. However, some conclusions (e.g., strong claims about adaptive convergence or plasmid transfer across lineages) go beyond the data, given the limitations of short-read sequencing and lack of phylogenetic inference.

Thank you for this thoughtful comment. To support our interpretation regarding convergent evolution, we have added a reference to a recent publication by Mas Fiol et al. (2024), in which convergent evolution among African *Y. pestis* strains has been reported.

The authors also understate the ambiguity around taxonomic assignment of outlier strains (e.g., Y. pseudotuberculosis-like genomes classified as Y. pestis).

We used these outlier strains to illustrate that traditional species identification based on biochemical tests can be inconclusive. Their inclusion in the study highlights the limitations of phenotypic classification methods and underscores the importance of genomic approaches for accurate taxonomic assignment.

**Are the limitations of the analysis clearly described?**
The authors do not sufficiently acknowledge several key limitations:

No long-read data for plasmid resolution or structural variation.

We acknowledge the limitation regarding the lack of long-read data for resolving plasmid structures and structural variation. The compatibility of genome assemblies generated using short- and long-read technologies remains a topic of active debate within the scientific community and would merit a dedicated comparative study. In this manuscript, we deliberately relied on short-read Illumina data to maintain consistency and avoid potential biases introduced by mixed sequencing platforms.

Small subgroup sizes for comparisons (e.g., Talas isolates).

We acknowledge the limited sample size for the Talas isolates. No specific conclusions were drawn based on this subgroup, as such comparisons would require additional statistical validation. These isolates were included to illustrate the broader range of genetic variability among *Y. pestis* strains from Central Asia.

Reliance on selected SNPs and parsimony trees rather than robust phylogenetic or population genetic approaches.

Please consider the following statements in the manuscript:

Lanes 454-458: „It should be noted that the dendrogram shown in Figure 3 should not be interpreted as a phylogenetic tree. The polymorphic sites were specifically selected to distinguish between sub-variants of the dominant MED biovar among the Y. pestis isolates. This explains why ANT, Talas, non-main, and Y. pseudotuberculosis strains are grouped together in the dendrogram, as the selected polymorphisms are not characteristic of these clades.”

614-618: „When a group of strains with a specific polymorphism is identified, researchers often, either explicitly or implicitly, assume that the group is phylogenetically derived from a recent common ancestor, overlooking the possibility that similar mutations could have arisen independently through convergent evolution in response to adaptation to the same hosts or similar environmental conditions.”

690-694: „The analysis of the distribution of different types of transposons and CRISPR-Cas elements on the chromosomes demonstrated the robustness of the method in identifying different lineages of Y. pestis and Y. pseudotuberculosis (Figure 4). Although the method is applicable for identifying distinct Yersinia clades [35, 71], it should not be used for phylogenetic inferences, as no computational models currently exist to translate differences in transposon distribution patterns into evolutionary distances.”

702-707: „The major discovery of this study is the demonstration of genetic diversity among the dominant MED Y. pestis isolates in Central Asia, revealing their division into desert and upland branches. It was shown that these groups are polyphyletic in terms of phylogeny, as adaptation to different mammalian hosts and environmental conditions has a greater influence than ancestral relationships between Y. pestis and Y. pseudotuberculosis isolates. While the identified polymorphisms may help determine the origin of an isolate, they are not suitable for phylogenetic inferences.”

No formal statistical or model-based testing of associations between genotype, host, or environment.

This question was addressed earlier.

Recommendation: A dedicated paragraph in the Discussion should explicitly list these analytical limitations and their implications for interpretation.

Additional text has been added to the Results and Discussion section to address this concern. We kindly ask the reviewer to check whether the revised version adequately acknowledges and discusses the relevant analytical limitations.

**Do the authors discuss how these data can be helpful to advance our understanding of the topic under study?**
Yes, in part.
The study contributes a valuable genomic dataset from a poorly sampled geographic region. The authors suggest that their polymorphism panel could be used for biovar identification and ecological surveillance. However, they could better articulate:

How these tools might integrate with global Y. pestis surveillance frameworks, or
How their SNPs or plasmid findings compare to those in outbreak strains from other continents.

Dear Reviewer, thank you for this insightful suggestion. We fully agree that the questions you raise are important. However, the current study is focused specifically on the Central Asian region, which is itself geographically large and includes multiple natural plague foci. Expanding our conclusions to a global context would require the inclusion of isolates from other regions, which was beyond the scope of this study. Without such data, broader comparisons or integration with global surveillance frameworks would remain highly speculative. Nonetheless, we acknowledge this as a direction for future research and have added a brief note to Conclusions (lines 732-737):

While this study focuses specifically on *Y. pestis* isolates from Central Asia, the genomic patterns observed, particularly those related to ecological adaptation and genetic variations, highlight the potential value of integrating regional datasets into broader surveillance frameworks. Future studies should include representative strains from other geographic regions to assess the universality of the identified polymorphisms. Such comparative analyses would be essential for aligning regional genomic surveillance efforts with global strategies for monitoring *Y. pestis* evolution and outbreak potential.

**Is public health relevance addressed?**
Minimally.
The manuscript briefly mentions zoonotic risk, ecological change, and the importance of surveillance in Kazakhstan, but does not connect this meaningfully to:

The risk of human outbreaks, or
How these findings might guide vector control, diagnostics, or cross-border health policy.

Recommendation: Strengthen the final paragraph of the Discussion to explicitly state how the findings could improve public health risk assessment, surveillance tools, or outbreak preparedness.

We acknowledge that the current manuscript does not focus on direct public health implications. Although the original collection includes numerous Y. pestis isolates from human patients and domestic animals, we intentionally excluded them from this study. Such data would require a dedicated investigation, ethical review, and a different analytical framework. This study is focused exclusively on Yersinia isolates from natural plague foci in order to better understand their ecological and evolutionary dynamics.

Nonetheless, we recognize the importance of situating our findings within a broader public health context and have added a brief statement to the end of the Conclusion mentioned above (lines 710-722).

# Editorial comments

In brief:

• Please articulate an explicit, testable hypothesis and streamline the Introduction to highlight the specific knowledge gap addressed.

• Descriptive SNP/parsimony analyses alone are insufficient. Add a statistically supported phylogeny and at least one population-structure or phylogeographic test linking genotype to ecology/host.

• Explain how strains were chosen from the national collection and acknowledge the inherent sampling imbalance when interpreting ecological patterns.

• Provide an explicit statement of BSL-3 approvals and regulatory compliance for work with Y. pestis.

• Improve figure resolution (vector graphics), split intermixed Results/Discussion text, and enforce consistent terminology for biovars, clades, and strain names.

• Correct Table 1 primer information; supply amplicon sizes, biovar specificity, and software versions/parameters for all tools.

All these issues were addressed in responds to the reviewers’ comments.

• Add a concise paragraph on how your SNP panel or plasmid findings could aid surveillance and explicitly list analytical limitations (e.g., lack of long-read data, small subgroup sizes).

Text was added to Conclusion, lines 710-722.

1) Please provide an Author Summary.

Author Summary was added

i) Please confirm (a) that you are the photographer of S2, or (b) provide written permission from the photographer to publish the photo(s) under our CC BY 4.0 license.

The microscopic photos were taken by the authors of the manuscript using MAGUS Lum 400L microscope. The text was added to the section Methods and the legend of this suppl. Figure was changed:

Supplemental Figure S2. Shapes of colonies of Y. pestis exemplified by strain 9_YP68_IM: A) after 12 hours; B) after 24 hours; and C) after 48 hours on Hottinger agar at 28°C; and D) after 24 hours on Hottinger agar supplemented with 1% blood hemolysate.

ii) Figure 1. Please (a) provide a direct link to the base layer of the map (i.e., the country or region border shape) and ensure this is also included in the figure legend; and (b) provide a link to the terms of use / license information for the base layer image or shapefile. We cannot publish proprietary or copyrighted maps (e.g. Google Maps, Mapquest) and the terms of use for your map base layer must be compatible with our CC BY 4.0 license.
Note: if you created the map in a software program like R or ArcGIS, please locate and indicate the source of the basemap shapefile onto which data has been plotted.

Text was added to the Methods (lines 190-193): Precise coordinates of the isolation sites were recorded and plotted on the map shown in Figure 1 using ArcMap 10.8 (https://desktop.arcgis.com/en/arcmap/), installed at the NCMO. The administrative boundaries on the map were obtained from the GADM database of Global Administrative Areas (version 2.8) accessible at https://gadm.org.

The reference to the GADM database was also added to the legend of Figure 1.

4) Please amend your detailed Financial Disclosure statement. This is published with the article. It must therefore be completed in full sentences and contain the exact wording you wish to be published.
1) State the initials, alongside each funding source, of each author to receive each grant. For example: "This work was supported by the National Institutes of Health (####### to AM; ###### to CJ) and the National Science Foundation (###### to AM)."
2) State what role the funders took in the study. If the funders had no role in your study, please state: "The funders had no role in study design, data collection and analysis, decision to publish, or preparation of the manuscript."
3) If any authors received a salary from any of your funders, please state which authors and which funders..
If you did not receive any funding for this study, please simply state: u201cThe authors received no specific funding for this work.u201d

Financial disclosure was added after the Acknowledgements:

This work was supported by the Minister of Science and Higher Education of the Republic of Kazakhstan; Grant AP19680079, "Study of molecular genetic features and variability of plague and tularemia strains in epidemiological surveillance of zoonoses" to AAA. The funder had no role in study design, data collection and analysis, decision to publish, or preparation of the manuscript.
