# Supplementary material for: Whole genome sequencing of Yersinia pestis isolates from Central Asian natural plague foci revealed the role of adaptation to different hosts and environmental conditions in shaping specific genotypes
Source: PLoS Negl Trop Dis. 2025 Sep 12;19(9):e0013533. doi: 10.1371/journal.pntd.0013533 (PMC12445494; doi:10.1371/journal.pntd.0013533)
Supplement: Attachment — Submitted filename: point-to-point-responses.docx [file pntd.0013533.s014.docx]

Reviewer #2: This manuscript analyses ~100 Yersinia pestis isolates from Central Asian plague foci using short-read WGS, targeted polymorphism panels, plasmid profiling and classical phenotyping. The dataset is valuable and the revision improves framing, biosafety statements, figure exports, and data availability. However, several core analytical and reporting issues remain.

Major points
Phylogenomic backbone: Provide a core-genome SNP maximum-likelihood tree (with support) for all isolates and use it to anchor ecological interpretations; the current diagnostic-SNP dendrogram is explicitly not a phylogeny.

Dear reviewer,

Thank you for this important comment. We acknowledge that the original Methods section may have been misleading due to the inappropriate use of the term “SNP.” In the revised version, this has been replaced with “polymorphism” or “polymorphic locus” for clarity.

The updated description is now provided in lines 449-467:

“Predicted genetic polymorphisms were filtered from indels and additionally by prediction quality parameters using the following thresholds: quality score (QUAL ≥ 30), depth of coverage (DP ≥ 10), mapping quality (MQ ≥ 40), mapping-quality zero fraction (MQ0F ≤ 0.1). The former filtering parameter was used to eliminate polymorphisms unique to individual strains, which may reflect sequencing errors. For each polymorphic locus repeatedly identified at least in 5 individual genomes, 81-bp context sequences surrounding the polymorphic site together with additional metadata regarding the location of the loci within protein-coding genes (CDS) of the reference genome (NC_006155.1) were recorded. The initial dataset comprises 7,007 selected polymorphic loci.

To verify variations in the selected polymorphic loci across the sequenced genomes of Y. pestis isolates, local BLASTN alignment was used to identify the locations 81 bp context sequences. BLASTN alignment parameters were set to identify matches of the same length as the queried context sequences, allowing no more than five mismatches (SNPs) per a 81 bp context sequence. This setting ensured the filtering out of false-positive alignments while retaining alignments with a few mismatches, which could result from assembly errors. During this process, the table of polymorphic loci was transformed into a 0/1 matrix, where 0 indicates the successful identification of a homologous context sequence in the assembled genome, and 1 indicates the absence of the reference context sequence in the assembled genome. The resulting matrix was filtered to remove loci where 0 or 1 states occurred in more than 94% of the genomes. The final 0/1 matrix contains 99 polymorphic sites suitable for genotyping (Suppl. Table S5).”

The current text made it clear that the polymorphic loci represent 81-bp highly variable regions, not single SNPs. Even with up to five substitutions per locus, these were considered the same variant. This design removed noise from numerous strain-specific SNPs but prevented presentation in pseudo-sequence format. The only suitable representation is as a 0/1 presence–absence matrix, which can be interpreted by certain parsimony algorithms such as Camin–Sokal, but not by sequence-based algorithms like maximum likelihood (LM).

As highlighted throughout the manuscript, phylogenetic inference was not an aim of this project. Our findings explicitly demonstrate that the diagnostic polymorphisms are largely homoplastic, i.e. arising from convergent adaptation to similar hosts and environments rather than shared ancestry, making them suitable for identification and surveillance, but completely unsuitable for phylogenetic inferences. Relevant clarifications appear in:

Lines 51-53 in Authors Summary: “These populations appear to reflect ecological adaptation rather than strict phylogenetic lineage, suggesting that convergent evolution plays a significant role in shaping *Y. pestis* diversity.”

Lines 168-173: “The aim was to identify novel marker mutations and to associate genetic polymorphisms with either ancestral inheritance or convergent evolution driven by adaptations to similar environmental conditions, mammalian hosts, or vector insects. The central hypothesis was that genetic variation in *Y. pestis* may correlate with host or environmental origin potentially obscuring clonal ancestry, thereby facilitating pathogen surveillance and offering new insights into its evolutionary dynamics.”

Lines 472-476: “It should be noted that the dendrogram shown in Figure 3 should not be interpreted as a phylogenetic tree. The polymorphic sites were specifically selected to distinguish between sub-variants of the dominant MED biovar among the *Y. pestis* isolates. This explains why ANT, Talas, non-main, and *Y. pseudotuberculosis* strains are grouped together in the dendrogram, as the selected polymorphisms are not characteristic of these biovars.”

Lines 631-633: “The aim of this study was to evaluate the potential of high-throughput sequencing technologies for monitoring the distribution and introgression of *Y. pestis* biovars and subclades associated with different environments and natural plague foci in Central Asia.”

Lines 647-651: “When a group of strains with a specific polymorphism is identified, researchers often, either explicitly or implicitly, assume that the group is phylogenetically derived from a recent common ancestor, overlooking the possibility that similar mutations could have arisen independently through convergent evolution in response to adaptation to the same hosts or similar environmental conditions.”

Line 713: “In this study, we were unable to identify polymorphisms with strong, unambiguous phylogenetic signals, …”

Lines 757-760 in Conclusion: “While the identified polymorphisms may help determine the origin of an isolate, they are not suitable for phylogenetic inferences. Researchers working with these pathogens should be cautious when inferring relationships between isolates based on a simple count of shared or differing SNPs and indels.”

Figure 3 capture: “The dendrogram of clusters of *Yersinia* isolates based on the 0/1 matrix of successful/unsuccessful BLASTN searches for sequences of polymorphic regions across the whole genome sequences of the isolates…”

Genome QC: Add a per-isolate table (genome size, N50/L50, contig count, %GC, mean depth) and state any exclusions.

The following was added:

Lines 347-353: “Whole-genome sequences were assembled using the in-house ILLAMINA pipeline and YPPA pipelines for assembly of chromosomes and plasmids, respectively [48, 49]. Each replicon (chromosome or plasmid) was represented by a single complete sequence without gaps. N’s in the sequences indicated nucleotide positions of known length that were characterized by low quality or ambiguous base calls. The GC content of the assembled chromosomes ranged from 47.5% to 47.6%. Genome assembly statistics, including assembly length, GC%, and mean sequencing depth (coverage) calculated for each replicon, are shown in Suppl. Table S3.”

Statistics: The random-forest classification of desert vs upland needs clearer methods (feature set, CV scheme, class balance) and proper p-value reporting (e.g., p < 0.001, not 0.0).

The revised text in lines 490-499 reads:

“The significance of segregation between the desert (n = 37) and upland (n = 47) variants of Y. pestis MED genomes was evaluated using the matrix of polymorphic loci (Suppl. Table S5) as the feature set. We applied the RandomForestClassifier and model_selection.cross_val_score functions from the Python scikit-learn library (version 1.7.0) in a 5-fold stratified cross-validation scheme, which preserved the original class proportions in each fold. The mean cross-validation accuracy was 0.9889, indicating that the probability of misclassifying isolates between the two environments based on the selected SNPs is about 1%. To further test robustness, we conducted a bootstrap analysis (1,000 permutations) in which clade labels were randomly shuffled, generating a null distribution of accuracies. The graphical output of the analysis is shown in Suppl. Figure S4. The observed accuracy exceeded all values from the null distribution, yielding a p-value < 0.001.”

Additionally, new Suppl. Fig. S4 with the graphical output of the bootstrap analysis was added.

Consider a simple AMOVA/Mantel/PERMANOVA to quantify genotype–ecology association.

Thank you very much for this insightful suggestion to perform additional statistical validation of genotype–ecotope associations and the intensity of cross-population introgressions. Text added:

Lines 307-314: “Additionally, PERMANOVA and Mantel tests for assessing the statistical significance of clade segregation were applied using the implementations provided in the stats.distance module of the scikit-bio package (v.0.7.0). An in-house Python 3 pipeline, yp_classifier, was developed for this project to perform all of the aforementioned statistical tests. The pipeline is available at https://zenodo.org/records/16875681.

Confidence interval (CI) of cross-population introgressions was calculated using the Wilson score interval [51], which is applicable for analyzing genotype-ecotope associations in microbiology [52] (Eq. 1).

$CI=\frac{p+pz^{2}\mp z\sqrt{\frac{p(1-5)}{n}+\frac{z^{2}}{4n^{2}}}}{1+\frac{z^{2}}{n}}$	Eq. 1.

where p is the observed proportion of introgressions; n is sample size; z = 1.96 for a 95% CI.”

Lines 499-503: “The same level of confidence, with a p-value of 0.001, was confirmed by the PERMANOVA and Mantel tests. All these tests were implemented in an in-house Python 3 script, available at https://zenodo.org/records/16875681. The matrix of 0/1 states of polymorphic loci (file example.csv), formatted for analysis by this program, can be found in the ‘input’ subfolder after downloading the program from the Zenodo repository.”

Lines 662-677: “This analysis revealed a clear separation of the 2.MED1 strains into two clusters, primarily isolated from desert and upland regions of Central Asia. The segregation significance was supported by the Random Forest classifier (accuracy = 0.9889), PERMANOVA test (F = 21.959), and Mandel test (r = 0.2707).

To estimate the intensity of introgression between subpopulations shown in Figure 3, the isolates were grouped by their sources of isolation from desert and upland plague foci (see Figure 1), irrespective of their genotype. The values of the segregation statistics decreased: classification accuracy = 0.7140, PERMANOVA F = 7.746, Mantel r = 0.1155, indicating a substantial level of cross-population introgression. The discordance rate p was estimated as the difference between genotype-based and ecotope-based classification accuracies, and this value was converted by Eq. 1 into a 95% CI (16%–34%) for an average introgression frequency. Introgressions from upland foci into desert foci outnumbered those in the opposite direction (Figure 3). Among 50 isolates from the desert foci, 16 (32%) carried genotypes characteristic of the upland regions, whereas only 4 of 34 isolates from the upland foci (11.8%) carried the desert-specific genotype. Estimation of the 95% CI using the Wilson score interval (Eq. 1) showed that the frequency of introgressions from upland regions into deserts ranged from 20.8% to 45.8%, whereas the reverse rate ranged from 4.7% to 26.6%.”

Reproducibility: List exact versions/parameters for Trimmomatic, SPAdes, Bowtie2, BCFtools, Prokka, RagTag (and seeds for ML).

Program versions were added in Methods as follows:

Trimmomatic v0.36, SPAdes v3.15.0, Bowtie2 v2.4.1, BCFtools/SAMtools v1.18, Prokka v1.13.3, RagTag v2.1.0, ML – not applicable.

Plasmid claims: The pCKF signal is interesting; include read-level evidence in Supplement (coverage plots; discordant pairs). If feasible, note any targeted validation.

Assembly statistics for the plasmid pCKF are now included in new Suppl. Table S3:

| **Genome**  42_YP10_UE | **Replicon** 3288_S10_pCKF | **Length** 5480 | **GC%** 38.52 | **Mean coverage** 226 | **Accession** SAMN47876487 |
| --- | --- | --- | --- | --- | --- |

This plasmid assembly statistic was also discussed in lines 568-570: “The depth coverage of the pCKF plasmid in strain 42_YP10_UE was comparable to that of pMT1 and pCD, with depths of 226, 196, and 194 reads per base, respectively.”

The mean coverage is >2× that of the chromosome. We analyzed the coverage plot using a 500 bp sliding window with a 100 bp step. The coverage distribution was uniform along the sequence, dropping only at the end of the reference sequence to as low as 38× in the last window. However, this drop is recovered when the reference sequence is swapped. Note that the ILLAMINA pipeline automatically removes consensus sequence regions with coverage below 10×.

We do not consider it necessary to include this uniform coverage plot as supplementary material.

Minor points
Tighten language and standardise terminology (biovar/clade/subvariant).

The term “biovar” is retained for MED, ANT, and ORI, while subdivisions are termed “clades” or “subclades.” “Subvariant” was replaced accordingly throughout. “Population” is used for 2.MED0.

Keep the pipeline graphic in Supplement; ensure all main figures have legible fonts/keys.

The pipeline diagram remains in the Supplement. All main figure fonts/keys have been adjusted for legibility.

Add a brief global context paragraph situating these lineages relative to established Y. pestis diversity.
End with a clearer public-health relevance statement (surveillance utility of markers, implications for risk assessment).

The following text was added or modified:

Lines 628-633 in Discussion: “Globally, *Y. pestis* diversity is structured into several established biovars associated with geographic foci and specific hosts. The separation of the pathogen population into distinct biovars may reflect both the ancient evolutionary history of the species and its adaptation to environmental conditions, as well as to the hosts and insect vectors. The aim of this study was to evaluate the potential of high-throughput sequencing technologies for monitoring the distribution and introgression of *Y. pestis* biovars and sub-clades associated with different environments and natural plague foci in Central Asia.”

Lines 749-754 in Conclusion: “The major discovery of this study is the demonstration of genetic diversity among the dominant MED *Y. pestis* isolates in Central Asia, revealing their division into desert and upland subclades. Within the broader framework, the desert and upland variants described here represent ecologically differentiated subclades of the same globally recognized MED biovar. The genomic differences identified between these subclades contribute to a more nuanced understanding of *Y. pestis* population structure and its ecological adaptation.”

Lines 761-773 in Conclusion: “The panel of *Y. pestis* genetic polymorphisms developed in this study provides a valuable tool for high-resolution surveillance of pathogens, particularly for distinguishing MED biovar strains into ecologically meaningful upland and desert branches. These polymorphisms, combined with the analysis of mobile genetic elements such as transposons, CRISPR-Cas systems, and plasmids including the concerning detection of the pCKF plasmid in a non-related isolate, can aid in tracking lineage distribution, potential host adaptation, and horizontal gene transfer across plague foci. Integration of these approaches into routine plague monitoring can enable rapid and accurate lineage identification, facilitating timely source attribution during outbreak investigations. This enhanced resolution supports more precise risk assessments in endemic areas by identifying ecologically specialized variants with differing potentials for host range expansion or virulence. The approach demonstrated here, grounded in the combination of whole-genome sequencing with robust statistical validation, can be readily incorporated into national and international plague surveillance frameworks, thereby strengthening preparedness and early-warning capabilities for the emergence or re-emergence of *Y. pestis* variants of public health concern.”

Data availability is strong; please also provide a pinned, isolate-level metadata CSV.

All metadata is provided in Suppl. Tables S1 and S3. To facilitate the use of this data, Suppl. Table S1 was converted from Word File to Excel Spreadsheet.
